# Soybean *GmHY2a* encodes a phytochromobilin synthase that regulates internode length and flowering time

**DOI:** 10.1093/jxb/erac318

**Published:** 2022-08-10

**Authors:** Zhirui Zhang, Suxin Yang, Qiushi Wang, Hui Yu, Beifang Zhao, Tao Wu, Kuanqiang Tang, Jingjing Ma, Xinjing Yang, Xianzhong Feng

**Affiliations:** Key Laboratory of Soybean Molecular Design Breeding, Northeast Institute of Geography and Agroecology, The Innovative Academy of Seed Design, Chinese Academy of Sciences, Changchun 130102, China; University of Chinese Academy of Sciences, Beijing 100049, China; Key Laboratory of Soybean Molecular Design Breeding, Northeast Institute of Geography and Agroecology, The Innovative Academy of Seed Design, Chinese Academy of Sciences, Changchun 130102, China; University of Chinese Academy of Sciences, Beijing 100049, China; Key Laboratory of Soybean Molecular Design Breeding, Northeast Institute of Geography and Agroecology, The Innovative Academy of Seed Design, Chinese Academy of Sciences, Changchun 130102, China; Key Laboratory of Soybean Molecular Design Breeding, Northeast Institute of Geography and Agroecology, The Innovative Academy of Seed Design, Chinese Academy of Sciences, Changchun 130102, China; Key Laboratory of Soybean Molecular Design Breeding, Northeast Institute of Geography and Agroecology, The Innovative Academy of Seed Design, Chinese Academy of Sciences, Changchun 130102, China; Key Laboratory of Soybean Molecular Design Breeding, Northeast Institute of Geography and Agroecology, The Innovative Academy of Seed Design, Chinese Academy of Sciences, Changchun 130102, China; University of Chinese Academy of Sciences, Beijing 100049, China; Key Laboratory of Soybean Molecular Design Breeding, Northeast Institute of Geography and Agroecology, The Innovative Academy of Seed Design, Chinese Academy of Sciences, Changchun 130102, China; Key Laboratory of Soybean Molecular Design Breeding, Northeast Institute of Geography and Agroecology, The Innovative Academy of Seed Design, Chinese Academy of Sciences, Changchun 130102, China; Key Laboratory of Soybean Molecular Design Breeding, Northeast Institute of Geography and Agroecology, The Innovative Academy of Seed Design, Chinese Academy of Sciences, Changchun 130102, China; University of Chinese Academy of Sciences, Beijing 100049, China; Key Laboratory of Soybean Molecular Design Breeding, Northeast Institute of Geography and Agroecology, The Innovative Academy of Seed Design, Chinese Academy of Sciences, Changchun 130102, China; University of Chinese Academy of Sciences, Beijing 100049, China; University of Auckland, New Zealand

**Keywords:** Flowering time, GmHY2a, internode length, phytochrome, phytochromobilin synthase, soybean

## Abstract

Plant height and flowering time are important agronomic traits that directly affect soybean [*Glycine max* (L.) Merr.] adaptability and yield. Here, the *Glycine max long internode 1* (*Gmlin1*) mutant was selected from an ethyl methyl sulfonate (EMS)-mutated Williams 82 population due to its long internodes and early flowering. Using bulked segregant analysis (BSA), the *Gmlin1* locus was mapped to *Glyma.02G304700*, a homologue of the Arabidopsis *HY2* gene, which encodes a phytochromobilin (PΦB) synthase involved in phytochrome chromophore synthesis. Mutation of *GmHY2a* results in failure of the de-etiolation response under both red and far-red light. The *Gmlin1* mutant exhibits a constitutive shade avoidance response under normal light, and the mutations influence the auxin and gibberellin pathways to promote internode elongation. The *Gmlin1* mutant also exhibits decreased photoperiod sensitivity. In addition, the soybean photoperiod repressor gene *E1* is down-regulated in the *Gmlin1* mutant, resulting in accelerated flowering. The nuclear import of phytochrome A (GmphyA) and GmphyB following light treatment is decreased in *Gmlin1* protoplasts, indicating that the weak light response of the *Gmlin1* mutant is caused by a decrease in functional phytochrome. Together, these results indicate that GmHY2a plays an important role in soybean phytochrome biosynthesis and provide insights into the adaptability of the soybean plant.

## Teaser

The longer internodes and earlier flowering of *Gmlin1*mutants are caused by mutations in GmHY2a, a phytochromobilin (PΦB) synthase that controls the phytochrome-mediated light response of soybean.

## Introduction

Soybean [*Glycine max* (L.) Merr.] is one of the most economically important leguminous seed crops globally, providing more than a quarter of the total protein in food and animal feed worldwide (Muramoto, 1999; Graham and Vance, 2003). Concomitant with economic development and population accretion, soybean demand is gradually increasing, requiring increased soybean yield (Liu *et al.*, 2020). Soybean adaptability and productivity are directly linked to plant height and flowering time, these important agronomic traits depend not only on endogenous genetic manipulation but also on environmental signals (Eshed and Lippman, 2019). When plants are grown under high-density conditions, the neighbouring vegetation absorbs red (R) light and reflects or transmits far-red (FR) light, which triggers a series of characteristic shade-avoidance syndromes (SASs), including the elongation of the hypocotyl (or stem) and petiole, the reorientation of the growth directions of the leaves or branches, and the acceleration of flowering (Carriedo *et al.*, 2016).

Plant perception of neighbour proximity or canopy shading is primarily mediated by the phytochromes, which are R- and FR-light photoreceptors (Liu *et al.*, 2021). Phytochromes exist in two photo-convertible forms: an inactive R-absorbing Pr form and an active FR-absorbing Pfr form (Li *et al.*, 2011). Under dark conditions, Pr form phytochromes are synthesized in the cytoplasm; following exposure to normal light (high R: FR), the Pr form phytochromes are photoconverted to the Pfr form and translocated to the nucleus (Franklin and Quail, 2010). The FR-rich conditions of vegetation shade shift the dynamic phytochrome equilibrium toward the inactive Pr form, leading to changes in the downstream signalling pathway, which subsequently alters plant traits and morphologies (Mawphlang and Kharshiing, 2017). Phytochrome apoproteins are encoded by a small multigene family (e.g. *PHYA* to *PHYE* in Arabidopsis). Phytochrome A (phyA), which is light labile, is the primary phytochrome in etiolated seedlings but is rapidly degraded to much lower steady-state levels upon transfer to light. In contrast, phyB**-**phyE are light stable in the Pfr form, and phyB is the predominant phytochrome regulating de-etiolation responses in R light (Franklin and Quail, 2010; Li *et al.*, 2011). Of the five phytochromes identified in Arabidopsis, phyB is the predominant negative regulator of SAS (Reed *et al.*, 1993). Active phyB triggers the phosphorylation of phytochrome interacting factors (PIFs), leading to their proteasome-mediated degradation. Under shade conditions, phyB inactivation stabilizes PIFs and allows them to bind and activate downstream targets, mostly auxin biosynthetic genes and cell wall-associated genes involved in promoting stem elongation (Liu *et al.*, 2021). Under deep canopy conditions, phyA has been shown to weaken auxin signalling to antagonize the SAS induced by phyB inactivation, thus preventing unnecessary shade-avoidance responses in severely light-deficient environments (Martínez-García *et al.*, 2014; Yang *et al.*, 2018).

In addition, the role of phytochromes in response to the changing light environments involves the perception of photoperiod, which has been well documented to provide important seasonal information that affects flowering and maturity (Franklin and Quail, 2010). The cultivation of soybean, which is a short-day (SD), photoperiod-sensitive plant, is limited to a narrow range of latitudes. Reducing photoperiod sensitivity to promote early flowering may allow the soybean to adapt to long-day (LD) conditions at higher latitudes. The homologues of soybean phyA, *E3* and *E4*, were identified as flowering and maturity loci using classical genetic approaches (Liu *et al.*, 2008; Watanabe *et al.*, 2009). A unique component and signalling pathway that regulates photoperiodic flowering is a specific *E1*-mediated regulatory pathway in soybean (Xia *et al.*, 2012). Under LD conditions, *E3* and *E4* promote the expression of *E1* and suppress the expression of the florigen genes *GmFT2a* and *GmFT5a* (*FLOWERING LOCUS T*), resulting in late flowering (Lin *et al.*, 2021). Loss of function of the *E3* and *E4* alleles leads to photoperiod insensitivity and earlier flowering (Xu *et al.*, 2013).

The photoactive holo-phytochromes consist of a PHY apoprotein covalently attached to a linear tetrapyrrole chromophore (phytochromobilin, 3*E*-PΦB) (Terry, 1997). The ability of given phytochromes to absorb R and FR light depends on the structural isomerization of PΦB (Legris *et al.*, 2019). The phytochrome chromophore is synthesized in the plastid from 5-aminolevulinic acid (ALA) via the heme branch of the tetrapyrrole biosynthetic pathway (Terry *et al.*, 1993). The first committed step in the synthesis of PΦB is the conversion of heme to biliverdin (BV) IXα by the enzyme heme oxygenase (Weller *et al.*, 1996). BV IXα is reduced to 3*Z*-PΦB by PΦB synthase (Weller *et al.*, 1997), isomerized to 3*E*-PΦB, and assembled into the functional holo-phytochrome protein. PΦB synthase is a ferredoxin-dependent BV reductase encoded by *HY2* (Kohchi *et al.*, 2001); the *HY1* gene encodes heme oxygenase (Muramoto *et al.*, 1999). Disruption of chromophore synthesis inactivates the entire phytochrome system because all phytochrome proteins bind to the same chromophore (Sawers *et al.*, 2002). Chromophore-deficient mutants, including Arabidopsis *hy1* and *hy2* (Parks and Quail, 1991; Kohchi *et al.*, 2001), tomato *yg-2* and *aurea* (Muramoto *et al.*, 2005), pea *pcd1* and *pcd2* (Weller *et al.*, 1996, 1997), and rice *se5* and *se13* (Izawa *et al.*, 2000; Yoshitake *et al.*, 2015), are characterized by impaired photomorphogenesis due to lack of light-reversible phytochrome. Characterization of the rice *se5* and *se13* mutants has demonstrated that the phytochrome chromophore makes significant contributions to the regulation of flowering time (Izawa *et al.*, 2000; Yoshitake *et al.*, 2015).

In this study, a forward genetic strategy was employed to analyse a mutant soybean variety, herein named *Glycine max long internode 1* (*Gmlin1*), which exhibited long internodes and early flowering. We characterized the *Gmlin1* mutant and identified *GmHY2a* as its associated candidate gene. We found that *GmHY2a* encodes a PΦB synthase and plays a role in the holo-phytochrome response to light. The genetic disruption of chromophore synthesis in *Gmlin1* mutants demonstrated here provides a framework for the specific inactivation of the entire soybean phytochrome system. In addition, our results help to clarify the role of phytochrome signalling in soybean development and highlight the potential agronomic importance of light-induced phenotypic plasticity.

## Materials and methods

### Plant materials

The soybean cultivar Williams 82 was obtained from the Chinese Academy of Agricultural Sciences (Beijing, China). *Gmlin1-1* and *Gmlin1-2* mutants were generated from the seeds of Williams 82 using ethyl methyl sulfonate (EMS)-induced mutagenesis and selected based on phenotype (long internodes and early flowering). Flowering time was measured at the R1 stage, which is defined as the time from emergence to the opening of the first flower (Bernard, 1971). Plant height and internode length were recorded at maturation (R8 stage), while the seeds per plant, pods per plant, seed weight per plant, and 100-seed weight were measured after harvesting. To purify the genetic background, the *Gmlin1-1* and *Gmlin1-2* mutants were backcrossed for four generations in an experimental field in Changchun, Jilin Province, China (43° 88ʹN, 125° 35ʹE).

### Bulked segregant analysis (BSA)

We used M2-seq (Zhou *et al.*, 2021) to identify candidate genes in the *Gmlin1-1* mutant. We collected leaves from 50 wild-type plants and 45 *Gmlin1-1* mutants in the M_2_ segregation generation. The DNA samples subsequently sequenced were extracted from the leaves using the Plant Genomic Kit (TIANGEN, China). The candidate genomic regions were identified via whole-genome sequencing, with a depth of approximately 30×, using an Illumina HiSeqX (Illumina Inc., San Diego, CA, USA). Single-nucleotide polymorphisms (SNPs) and small indels were calculated between the wild-type and mutant bulked DNA samples by aligning the sequence reads of individual bulked DNA samples to the *Glycine max Wm82.a2.v1* reference genome (https://phytozome-next.jgi.doe.gov/). SNPs and indels were filtered to identify the candidate genes following Zhou *et al.* (2021).

The *Gmlin1-2* mutants were backcrossed with Williams 82 to generate the BCF_2_ segregating population for re-sequencing. DNA sequences from 50 F_2_ individuals expressing the wild-type phenotype and 50 F_2_ individuals expressing the *Gmlin1-2* phenotype were bulked into two pools. Candidate genomic regions were identified using a QTL-seq method developed for F_2_ population sequences (Takagi *et al.*, 2013). A genomic region with delta SNP >0.5 was selected as the candidate area. All sequences are available from the BIG Data Center (https://bigd.big.ac.cn/gsa/index.jsp) under accession number CRA005967.

### Vector construction and complementation testing

The full-length *GmHY2a* gene sequences, about 7.5 kb long, as well as the ~3 kb native promoter regions, were amplified from Williams 82 using overlapping PCR. The PCR amplicons were inserted into the binary vector pCAMBIA3301T between the restriction endonuclease sites *Sac*I and *Xma*I to generate the *GmHY2apro:GmHY2a* plasmid. The *GmHY2apro:GmHY2a* recombinant plasmid was introduced into *Agrobacterium tumefaciens* strain EHA105 and transformed in the *Gmlin1-1* mutant for a complementation test following *Agrobacterium*-mediated transformation as previously described (Yamada *et al.*, 2010). The primers used are listed in Supplementary Table S1.

### Phylogenetic analysis and conserved protein motif searching

Homologues of GmHY2a were identified by searching for the full-length amino acid sequence in the Phytozome proteome database (https://phytozome-next.jgi.doe.gov/) and the NCBI database (https://www.ncbi.nlm.nih.gov/) using the BLASTP program. The identified homologues were aligned with GmHY2a using ClustalW as implemented in MEGA7 (Kumar *et al.*, 2016). To analyse the interspecific functional conservation and phylogenetic relationships of the HY2 protein, neighbour-joining phylogenetic tree was then constructed based on alignment using MEGA7 with 1000 bootstrap iterations. Conserved protein motifs were identified using MEME (http://meme-suite.org/tools/meme) (Bailey *et al.*, 2009), with the maximum number of motifs to be found set to 5.

### Gene duplication and expansion analysis

We performed a synteny conserved block analysis to investigate the gene duplication pattern of the three copies of *GmHY2* in soybean. Intraspecific synteny blocks were detected using the MCScanX algorithm (Wang *et al.*, 2013a). Sequences of homologous genes were identified using BLASTP with an E-value cut-off of 1e-5 and other parameters set to default or recommended values. Non-synonymous substitutions per non-synonymous site (Ka) and synonymous substitutions per synonymous site (Ks) values for the homologues were calculated using TBtools (Chen *et al.*, 2020). The time (T) since each duplication event was calculated using the equation T=Ks/2E, where the mean synonymous substitution rate (E) for soybean was set to 6.1 × 10^−9^ substitutions per synonymous site per year (Lynch and Conery, 2000; Lavin *et al.*, 2005).

### RNA isolation and real-time quantitative PCR (RT-qPCR) analysis

To analyse the expression patterns of the *GmHY2* genes, total RNA was isolated from multiple soybean tissues, including leaves, the stem apex (SA), stems, roots at the V1 stage (first trifoliolate), flowers at the R1 stage (beginning of flowering), 2 cm pods at the R3 stage (beginning pod), and seeds at 20 days after pollination, using TRNzol universal reagent (TIANGEN Biotech Co., Beijing, China) following the manufacturer’s instructions. RNA concentration and quality were determined using a NanoDrop spectrophotometer (P330; Implen, Germany). First-strand cDNA was synthesized from 2 μg of total RNA using the TransScript One-Step gDNA Removal and cDNA Synthesis SuperMix with Anchored Oligo(dT)_18_ primer (TransGen Biotech, Beijing, China), following the manufacturer’s instructions. The cDNA was diluted to 100 ng/μl in sterile water, and 2 μl diluent was used as the template for RT-qPCR. RT-qPCR was performed using 2×RealStar Green Fast Mixture (GenStar, Beijing, China) on a Stratagene Mx3005P Sequence Detection System (Stratagene, La Jolla, CA, USA) following the manufacturer’s instructions. Expression levels were quantified using three biological replicates. The differences between groups were calculated with the 2^-ΔΔCt^ method. Relative expression levels were calculated using *Actin 11* (*Glyma.18G290800*) as an internal control (Zhang *et al.*, 2020). The primers used for RT-qPCR are listed in Supplementary Table S1.

### Subcellular localization analysis

To localize the GmHY2a protein, a 1-kb coding region of *GmHY2a* was amplified from Williams 82 and cloned into an entry vector (pDonor/zeo; Invitrogen, USA) using Gateway BP clonase II (Invitrogen, USA) recombination, following the manufacturer’s instructions. The correct Entry Clone was introduced into the destination vector pENSG-YFP using Gateway LR clonase II (Invitrogen, USA) recombination (Wenkel *et al.*, 2006). The coding region of *RbcS* (encoding a protein marker of chloroplast stroma in Arabidopsis) was amplified from Arabidopsis (Col-0) and infused into the binary vector pCAMBIA1300-mCherry. The *35S*:*YFP*-*GmHY2a* and *35S*:*AtRbcS*-mCherry plasmids were introduced into Arabidopsis (Col-0) mesophyll protoplasts as previously described (Yoo *et al.*, 2007).

To localize the GmPHYA and GmPHYB proteins, the coding sequences of *GmPHYA* and *GmPHYB* were amplified from Williams 82 and cloned into the pA7-YFP vector between the restriction endonuclease sites *Xma*I and *Sal*I to generate the *35S:GmPHYA*-*YFP* and *35S*:*GmPHYB*-*YFP* plasmids, respectively. The two plasmids were transformed into soybean mesophyll protoplasts from Williams 82 and the *Gmlin1-1* mutant. Soybean mesophyll protoplasts were prepared, transfected, and cultured as previously described (Xiong *et al.*, 2019). The transformed protoplasts were kept in the dark or illuminated with white light (100 μmol m^2^ s^−1^) for 10 min before observation under a fluorescence microscope (C2; Nikon, Japan). YFP fluorescence was visualized using excitation and emission wavelengths of 488 and 500–550 nm, respectively. mCherry fluorescence was visualized using excitation and emission wavelengths of 561 and 570–1000 nm, respectively. Chloroplast autofluorescence was visualized using excitation and emission wavelengths of 405 and 417–477 nm, respectively. The primers used to construct vectors for subcellular localization are listed in Supplementary Table S1.

### Luciferase complementation imaging (LCI) assays

To test the interaction between GmHY2 and GmFd2, LCI assays were performed in 4-week-old *Nicotiana benthamiana* leaves. The coding regions of *GmHY2a* and *GmHY2b* were fused with the pCAMBIA1300-NLUC vector and *GmFd2* was fused with the pCAMBIA1300-CLUC vector between the restriction endonuclease sites *Kpn*I and *Sal*I (Zhou *et al.*, 2018). The recombinant plasmids were introduced into *Agrobacterium tumefaciens* strain EHA105 and co-infiltrated into *Nicotiana benthamiana* leaves as described previously (Zhou *et al.*, 2018). LUC activity was analysed using chemiluminescence imaging (4600SF; Tanon, China) after infiltration for 48 h. The primers used for the LCI assay are listed in Supplementary Table S1.

### Photomorphogenic assays and end-of-day FR (EOD-FR) treatment

Photomorphogenic assays were performed as described previously (Izawa *et al.*, 2000; Liu *et al.*, 2008), with few modifications. Williams 82, *Gmlin1-1*, *Gmlin1-2*, and the transgenic complementation plants were grown at 25 °C in darkness, or under continuous R, FR, blue, or white light conditions in a multicolour LED incubator (ZDN-1000; YangHui, China). Hypocotyl length was measured 7 days after sowing. For EOD-FR treatments, plants were grown in a multicolour LED incubator with a 16 h light/8 h dark cycle under normal white light (WL, 100 μmol m^2^ s^−1^). At 3 days after emergence, half of the seedlings were treated with FR light (14 μmol m^2^ s^−1^) for 30 min at the end of the light period for 4 days before measurements were taken. Control seedlings were grown under continuous normal WL conditions. To measure the expression levels of SAS-related genes, seedling hypocotyl tissues were harvested 1 h after the end of the final EOD-FR pulse on day 7.

### Photoperiodic transfer treatment

Photoperiodic reciprocal transfer experiments were performed following the analytical model previously described (Ellis *et al.*, 1992). Two photoperiods were established: a LD photoperiod of 16 h light/8 h dark, and a SD photoperiod of 12 h light/12 h dark. The growth chambers were kept at a constant temperature of 25 °C. A tray of 15 plants was considered a block, and each transfer treatment was assigned to one block. The trays were periodically moved randomly around the growth chambers to reduce the impact of microclimatic conditions. Transfers were made at 5, 10, 15, 20, 25, 30, 35, and 40 days after emergence (DAE). Control plants were continuously grown under LD or SD conditions. Once plants had been transferred, they were maintained in the new growth chamber. Time to first flower (R1) (Fehr *et al.*, 1971) was noted for each plant. We performed a segmented linear regression analysis of the data obtained through the above transfer experiments with OriginPro 8.5. To analyse the expression patterns of flowering and photoperiodic genes, Williams 82 and the *Gmlin1-1* mutant were grown in a growth chamber under SD or LD conditions at 25 °C; fully expanded trifoliate leaves from 15 individual plants were collected and pooled every 4 h starting at dawn at 15 days after emergence (DAE).

## Results

### Phenotypic characterization of the soybean *Gmlin1* mutant

**Fig. 1.**
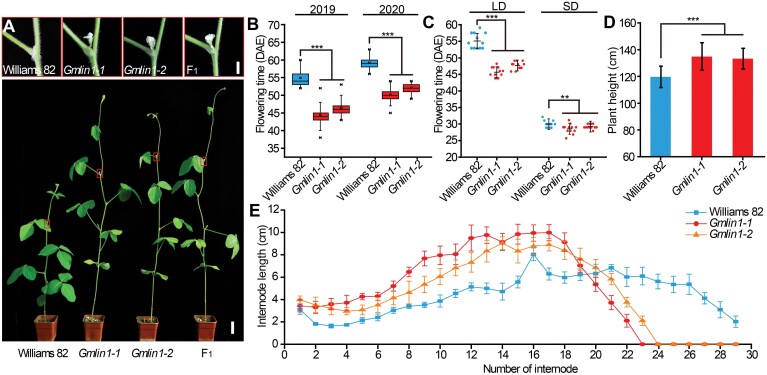
The phenotypes of the soybean wild-type (Williams 82) and *Gmlin1* mutants. (A) Phenotypes of Williams 82, the two *Gmlin1* mutants, and the F_1_ hybrid plant (*Gmlin1-1*×*Gmlin1-2*) at the flowering stage, grown in a growth chamber. Plants were grown under a 16 h light/8 h dark cycle. Scale bar=5 cm. The upper inset boxes are magnifications of the boxed areas in the lower image and show flower detail. Scale bar=1 cm. (B) Flowering times of Williams 82 and the two *Gmlin1* mutants grown under natural conditions in the field at Changchun, China (May to August in 2019 and 2020). (C) Flowering times of Williams 82 and the two *Gmlin1* mutants grown under artificial LD (16 h light/ 8 h dark) or SD (12 h light/ 12 h dark) conditions. (D) Plant heights of Williams 82 and the two *Gmlin1* mutants at maturity. (E) Internode lengths of Williams 82 and the two *Gmlin1* mutants at maturity. All data shown are means ±SD (at least 20 plants). Asterisks indicate statistically significant differences (**P<0.01, ***P<0.001, Student’s *t*-test).

Compared to the wild-type soybean cultivar (Williams 82), the EMS-induced soybean mutants *Gmlin1-1* and *Gmlin1-2* were significantly taller and flowered significantly earlier (Fig. 1A). In the field and under artificial LD conditions (16 h light/8 h dark), the *Gmlin1-1* and *Gmlin1-2* mutant lines flowered about 10 d earlier than Williams 82 (Fig. 1B, C). Even under the relatively extreme SD conditions (12 h light/12 h dark), the *Gmlin1-1* and *Gmlin1-2* mutants flowered 1 day earlier than Williams 82: a small, but statistically significant (*P*<0.01), difference (Fig. 1C). At maturation, the main stems of the field-grown *Gmlin1-1* and *Gmlin1-2* mutants were about 15 cm taller than those of Williams 82 (Fig. 1D). This difference in plant height was reflected in the longer internodes of the *Gmlin1-1* and *Gmlin1-2* mutants; the *Gmlin1* mutants had fewer internodes on the main stem (Fig. 1E). Genetic allelic tests were performed by crossing *Gmlin1-1* with *Gmlin1-2*. The phenotypes of the intercrossed F_1_ hybrids were similar to those of their parents (Fig. 1A). Sequencing analysis indicated the sites mutated in the parent mutants were heterozygous in the F_1_ plants (Supplementary Fig. S1), suggesting that *Gmlin1-1* and *Gmlin1-2* were allelic to each other and that both phenotypes were controlled by the same gene.

### Cloning and identification of the candidate gene associated with the *Gmlin1* mutants

**Fig. 2.**
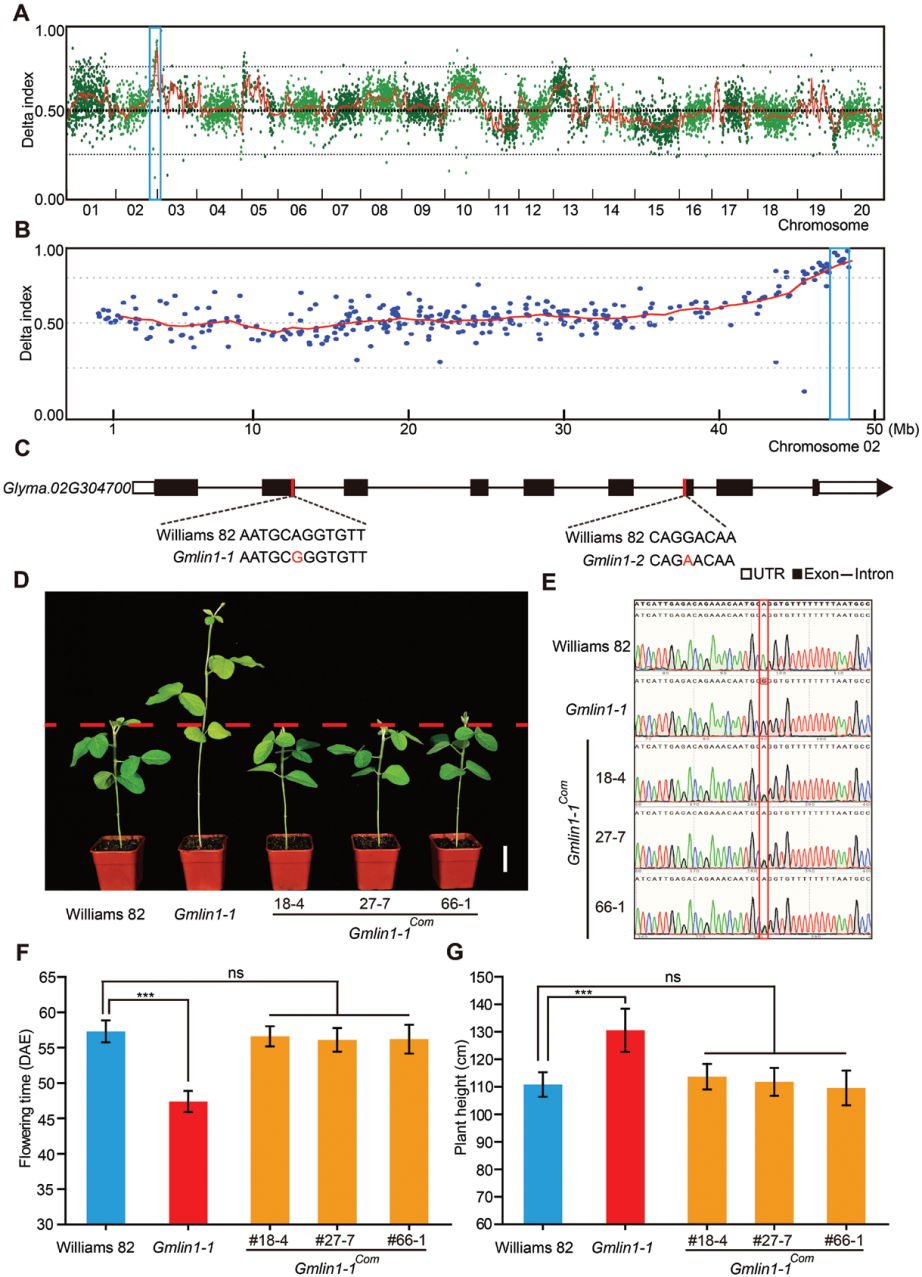
Bulked segregant analysis (BSA)-based localization of the candidate gene and functional complementation test. (A, B) Delta SNP indexes of (A) all chromosomes and (B) chromosome 02 of the F_2_ population of *Gmlin1-2*. (C) Genomic structure of the *Glyma.02G304700* gene. The red vertical lines represent the sites of the two allelic mutations. (D) Phenotypes of Williams 82, the *Gmlin1-1* mutant, and three independent complementary transgenic plants (*Gmlin1-1*^Com^) with the *Gmlin1-1* mutant background. Plants were grown in a growth chamber under a 14 h light/10 h dark cycle. Scale bar=5 cm. (E) Sequence analysis of the *Glyma.02G304700* gene showing the transgenic events. The red box represents the mutation site of the *Gmlin1-1* mutant. Two product peaks can be seen in the transgenic plants corresponding to both the wild-type and the mutant sequences. (F) Flowering times and (G) plant heights of Williams 82, the *Gmlin1-1* mutant, and the transgenic complementation lines. Plants were grown in the field at Changchun, China (May to August 2021). All data shown are means ±SD (at least 10 plants). Asterisks indicate statistically significant differences (***P<0.001, Student’s *t-*test). ns, non-significant difference.

To identify the candidate gene of *Gmlin1* mutant, BSA was performed on the *Gmlin1-1* M_2_ segregation population (Supplementary Fig. S2) and the *Gmlin1-2* backcross F_2_ (BCF_2_) segregating population (Fig. 2A, B). Of the 157 plants in the *Gmlin1-1* M_2_ population, 45 exhibited the *Gmlin1* mutant phenotype, indicating that this phenotype was consistent with the 3:1 segregation ratio of a single recessive nuclear gene (*X*^*2*^*=*1.228; *P*>0.05). After filtering out all undesirable variations, we screened for the most likely causal variant of *Gmlin1-1*. The likely variant was mapped to *Glyma.02G304700* based on delta SNP values between the wild-type pool and the mutant pool (Supplementary Fig. S2). Of the 248 BCF_2_ plants produced by backcrossing *Gmlin1-2* with Williams 82, 58 showed the *Gmlin1* mutant phenotype, which again corresponded to the expected 3:1 segregation ratio for a single recessive gene (*X*^2^=0.344; *P*>0.05). After filtering, we identified 6872 SNPs between the mutant and the wild-type pools (Fig. 2A). The statistically significant peak with the highest delta SNP value was located in the 3.1 Mb region between 45.5 Mb and 48.6 Mb on chromosome 02 (Fig. 2B). *Glyma.02G304700*, the candidate gene of *Gmlin1-1* identified above, was located in this region.

In the *Gmlin1-1* mutant, there was a single-nucleotide substitution (A to G at 1550 bp) in the second exon of *Glyma.02G304700* (Fig. 2C), resulting in two forms of mis-splicing (Supplementary Fig. S3). One mis-splice was a 41 bp intron insertion in the coding sequence immediately following the mutation, which caused a frameshift resulting in a premature stop codon and the encoding of a truncated protein. The other mis-splice was a 41 bp intron insertion in the coding sequence and the loss of exon 3, shifting the reading frame of the processed mRNA. The *Gmlin1-2* mutant had a single-nucleotide substitution (G to A at 5862 bp) in exon 7 of the *Glyma.02G304700* gene (Fig. 2C), which caused the non-synonymous substitution of Gly255 to Glu255 in the encoded protein.

To validate the candidate gene, a genetic complementation test was performed to confirm that these mutations produced the *Gmlin1* mutant phenotype. We obtained 30 independent positive transgenic lines in the *Gmlin1-1* background in the T_0_ generation; 24 of these transgenic lines had reverted to the wild-type phenotype in the T_1_ generation. Three independent complementation transgenic lines were selected for characterization analysis (Fig. 2D). Genetic analysis verified that transgenic plants in the *Gmlin1-1* background carried both the wild-type and *Gmlin1-1* mutant genotypes (Fig. 2E). We statistically analysed the flowering times and heights of the T_2_ generation transgenic plants grown in the field. In contrast to the *Gmlin1-1* mutant phenotype and similar to Williams 82, the flowering time of the three complementation transgenic lines was about 54 days, and the plant height of these lines was 110 cm at maturity (Fig. 2F, G). These results indicated that *Glyma.02G304700* was indeed the candidate gene.

### Conservation and duplication of *GmHY2* in soybean

**Fig. 3.**
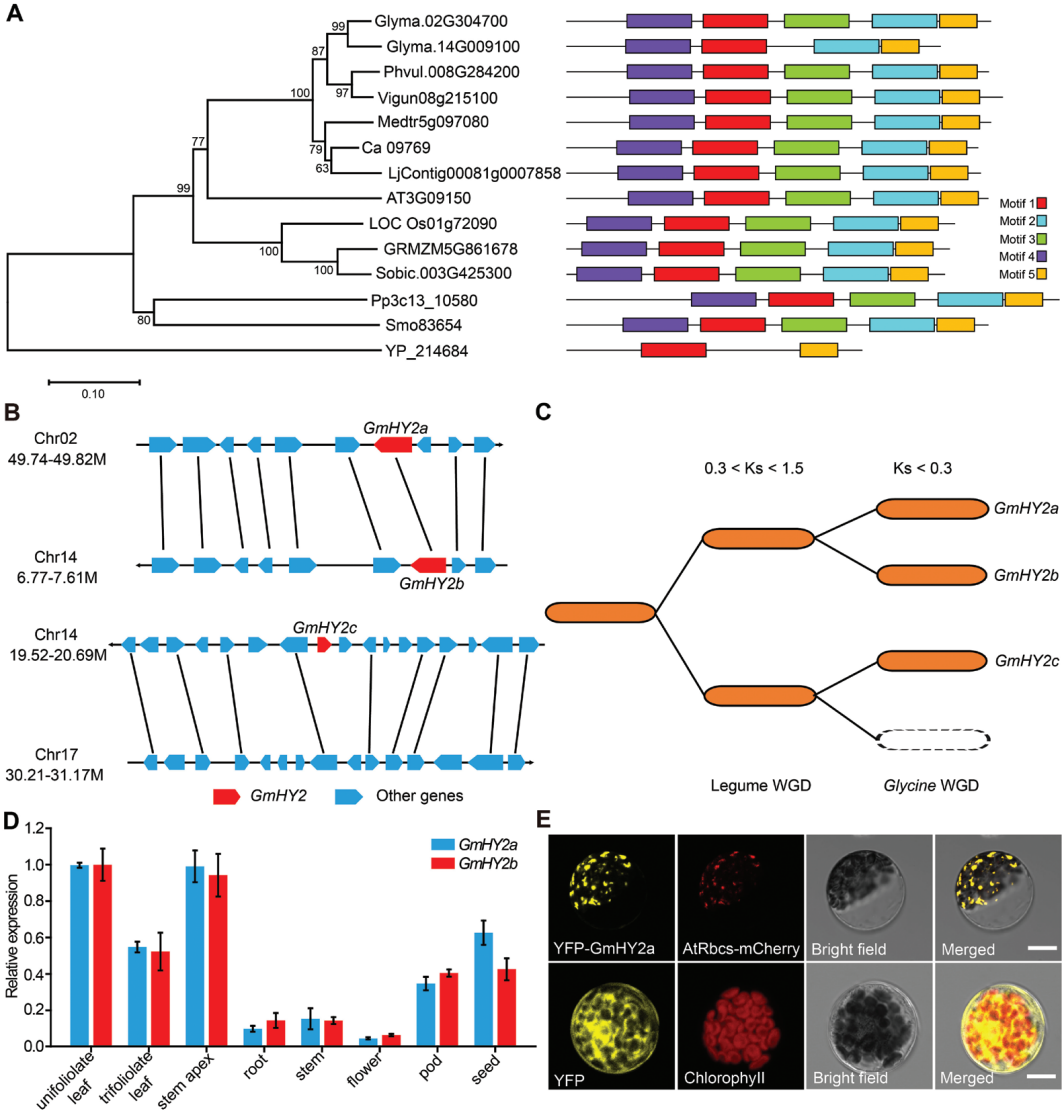
Functional analysis of GmHY2 and its homologues. (A) Phylogenetic analysis and motif comparisons among GmHY2a (Glyma.02G304700) and GmHY2b (Glyma.14G009100) from *Glycine max* and its homologues from *Phaseolus vulgaris*, *Vigna unguiculata*, *Medicago truncatula*, *Cicer arietinum*, *Lotus japonicus*, *Arabidopsis thaliana*, *Oryza sativa*, *Zea mays*, *Sorghum bicolor*, *Physcomitrella patens*, *Selaginella moellendorffii*, and *Prochlorococcus* phage. The five conserved motifs are represented by coloured boxes. (B) Syntenic relationships among homologous blocks carrying the *GmHY2* sequences. The red blocks represent *GmHY2*, and the blue blocks represent other genes around *GmHY2*. Solid black lines connect homologous gene pairs. (C) The evolutionary model for *GmHY2*-containing genomic blocks over the course of soybean genome evolution. 0.3<Ks<1.5 represents legume WGD, Ks<0.3 represents *Glycine* WGD. (D) Relative expression levels of *GmHY2a* and *GmHY2b* in various tissues of Williams 82. All data shown are means ±SD. (E) Subcellular localization of GmHY2a in Arabidopsis protoplasts. Free YFP was used as the control. Scale bars=10 μm.

Bioinformatics analysis indicated that the *Glyma.02G304700* gene encoded a phytochromobilin synthase (PΦB synthase), with a ferredoxin-dependent biliverdin reductase (FDBR) domain. The alignment of known homologous sequences indicated that the mutations of the candidate gene in *Gmlin1-1* and *Gmlin1-2* were both located in the FDBR structural domain and demonstrated that these sites were highly conserved across multiple plant species (Supplementary Fig. S4). As sequence comparisons showed that the candidate gene was a homologue of the Arabidopsis *HY2* (65% amino acid identity), we named the *Glyma.02G304700* gene *GmHY2a*. *Glyma.14G009100* (*GmHY2b*) and *Glyma.14G136300* (*GmHY2c*) genes are likely *GmHY2a* homologues in the soybean genome (*Glycine max Wm82.a4.v1*). An exon is lost in *GmHY2b* due to the exon skip in the coding sequence (Supplementary Fig. S5A). *GmHY2c* might be a pseudogene that had a truncated open reading frame length of 423 bp (Supplementary Fig. S4), and its expression level was predicted to be extremely low in the transcriptome database phytozome (https://phytozome-next.jgi.doe.gov/). Phylogenetic analysis of 14 homologues of the HY2 protein from 13 species showed that homologues of HY2 were present in a wide variety of photosynthetic organisms, including algae, basal vascular plants, monocotyledons, and dicotyledons, indicating that HY2 is highly conserved across plants. As expected, soybean GmHY2 was closest to its homologues from other plants in the Leguminosae (Fig. 3A). Across these 14 HY2 proteins, MEME analysis identified five significant motifs; GmHY2b lacked conserved motif 3 compared to GmHY2a and other higher plants (Fig. 3A).

Synteny analysis revealed that *GmHY2a* and *GmHY2b*, not *GmHY2c*, were likely duplicated genes in soybean (Fig. 3B). Using the 1 Mb region on chromosome 14 around *GmHY2c*, we found that some sequences flanking *GmHY2c* had a syntenic relationship with those on chromosome 17, suggesting that chromosome 17 was the most likely origin of *GmHY2c*. However, the *GmHY2c* homologue was lost from the homologous region of chromosome 17 (Fig. 3B).

Estimations of the possible duplication times of the three *GmHY2* genes based on the average synonymous substitution rate (*Ks*) for the homologous block suggested that *GmHY2a* and *GmHY2b* were duplicated about 11.15 Mya, *GmHY2c* and the corresponding region of chromosome 17 were duplicated about 12.4 Mya, corresponding to a *Glycine*-lineage specific whole genome duplication (WGD) about 13 Mya (Wang *et al.*, 2013b). The *GmHY2c* and *GmHY2a*/*GmHY2b* duplication events occurred about 36.37 Mya and 31.63 Mya, respectively (Fig. 3C; Supplementary Table S2). These latter two events were associated with an early legume WGD about 58 Mya, before the duplication of *GmHY2a* and *GmHY2b*. Gene duplication and expansion analysis suggested that the soybean *GmHY2* gene has undergone at least two duplication events: the legume duplication and the *Glycine*-lineage specific duplication. *GmHY2c* may have lost its function gradually after the legume duplication event and was not duplicated in the *Glycine*-lineage specific duplication event.

### Expression patterns and subcellular localization of GmHY2

Relative expression analysis showed that both *GmHY2a* and *GmHY2b* were constitutively expressed in all tested tissues (leaves, SAs, stems, roots, flowers, pods, and seeds), with the highest levels of expression found in the leaves and stem apex (Fig. 3D). The similar expression patterns of the two genes implied that both genes have normal transcription levels in soybean. However, the conserved sequences deleted in the FDBR domain of GmHY2b might affect the function of the protein (Supplementary Fig. S5B).

As a ferredoxin-dependent biliverdin reductase, HY2 requires ferredoxin (Fd2) as an electron donor in order to catalyse the substrate BV IXα (Chiu *et al.*, 2010). LCI assays in *N. benthamiana* leaf cells showed that both of the GmHY2 homologues (GmHY2a and GmHY2b) interacted with GmFd2 (Supplementary Fig. S5C). However, differences in LUC intensity indicated that the interaction between GmHY2a and GmFd2 was significantly stronger than that between GmHY2b and GmFd2 (Supplementary Fig. S5C). This result suggested that GmHY2 and GmFd2 interacted and that the loss of exon in GmHY2b affected the function of GmHY2. We speculated that the weak interaction suggests the GmHY2b proteins might have redundant functions. We thus speculated that GmHY2a plays an important role in the soybean chromophore biosynthesis pathway.

To determine the subcellular location of GmHY2a, we fused the yellow fluorescent protein (YFP) to the coding region of *GmHY2a* under the control of the 35S promoter, and then transferred the fusion protein to Arabidopsis protoplasts. In Arabidopsis protoplasts, YFP fluorescence signals, corresponding to the *35S*:*YFP*-*GmHY2a* fusion protein, were observed in a punctate pattern inside the chloroplasts (Fig. 3E). When YFP-GmHY2a was transiently co-expressed with AtRbcS (a marker protein of the chloroplast stroma in Arabidopsis) fused with mCherry (a red fluorescent protein), the yellow and red fluorescent signals were merged within the chloroplasts (Fig. 3E), demonstrating that GmHY2a was a chloroplast-targeted protein, similar to Arabidopsis HY2 (Kohchi *et al.*, 2001).

### The phytochrome-mediated light response was disrupted in *Gmlin1* seedlings

**Fig. 4.**
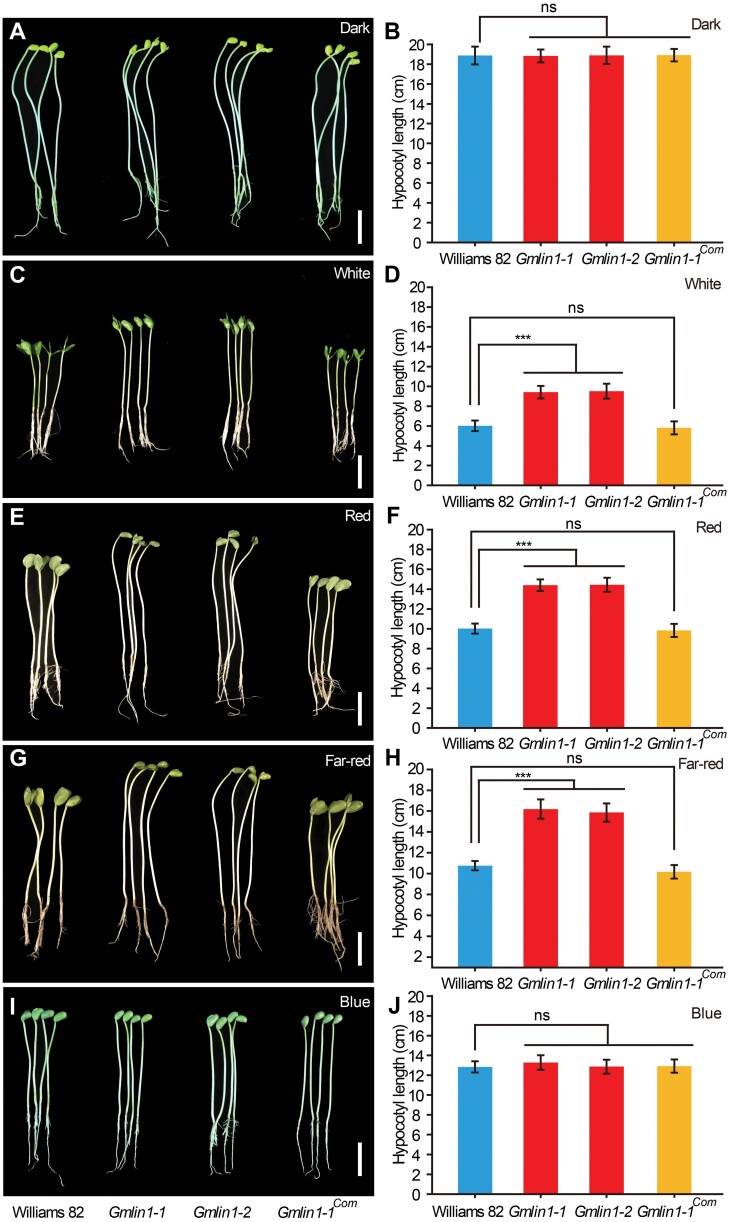
Phytochrome responses of Williams 82 and the *Gmlin1* mutants. Phenotypes and the corresponding hypocotyl lengths of Williams 82, the two *Gmlin1* mutants, and the *Gmlin1-1* complementation plants after 7 days of growth (A, B) in the dark, (C, D) under white light (100 μmol m^−2^ s^−1^), (E, F) under red light (31 μmol m^−2^ s^−1^), (G, H) under far-red light (14 μmol m^−2^ s^−1^), and (I, J) under blue light (63 μmol m^−2^ s^−1^). Scale bars=2 cm. Hypocotyl lengths are means ±SD (n=10). Asterisks above the graphs indicate statistically significant differences (***P<0.001, Student’s *t*-test). ns, non-significant difference.

To test the response of the *Gmlin1* mutants to light signals, we analysed the inhibitory effects of different light conditions on hypocotyl length, which is considered a standard test of light-responsiveness (Sawers *et al.*, 2002; Hu *et al.*, 2021). When grown in darkness, Williams 82, the *Gmlin1* mutants, and the transgenic complementation seedlings expressed similar etiolation phenotypes and had equally long hypocotyls (Fig. 4A, B). Under white, R, and FR light, Williams 82 seedlings and the transgenic complementation seedlings exhibited normal de-etiolation responses, whereas *Gmlin1* mutants exhibited significantly elongated hypocotyls and small cotyledons, suggesting that the sensitivity of the de-etiolation response to R and FR light in the mutant seedlings was reduced (Fig. 4C-H). However, under blue light, the phenotypes and hypocotyl lengths of the *Gmlin1* mutants were similar to those of Williams 82 (Fig. 4I, J). Thus, the *Gmlin1* mutants exhibited a lack of responsiveness to both R and FR light, suggesting that the phytochrome-mediated light responses were weakened in these mutants.

### Mutation in GmHY2a affected the nuclear import of GmphyA and GmphyB

**Fig. 5.**
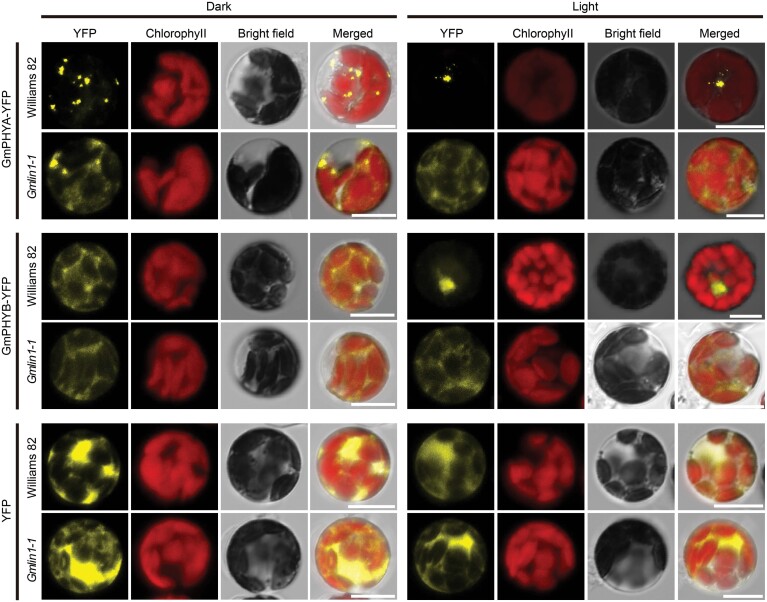
Subcellular localization of soybean phytochrome A and phytochrome B. GmPHYA-YFP and GmPHYB-YFP were expressed in soybean mesophyll protoplasts from Williams 82 and the *Gmlin1-1* mutant. YFP fluorescence appears yellow, while chloroplast autofluorescence appears red. Merged images show the combination of the three channels (YFP fluorescence, chloroplast autofluorescence, and bright field). Scale bars=10 μm.

The insensitivity of the *Gmlin1* mutants to R and FR light suggested that the mutation in GmHY2a might affect the photoconversion of phytochromes. To test this, GmPHYA and GmPHYB were fused with YFP and expressed in mesophyll protoplasts prepared from Williams 82 and the *Gmlin1-1* mutant. In the dark, GmPHYA-YFP and GmPHYB-YFP were mostly localized in the cytoplasm of both Williams 82 and *Gmlin1-1* protoplasts. Under light exposure, GmPHYA-YFP and GmPHYB-YFP were found in the nucleus of the Williams 82 protoplast, suggesting that light exposure initiated the nuclear transport of GmPHYA and GmPHYB. However, in the light-exposed *Gmlin1-1* protoplasts, GmPHYA-YFP and GmPHYB-YFP primarily remained in the cytoplasm, suggesting that phytochromes could not be effectively imported into the nucleus in the *Gmlin1-1* mutant (Fig. 5). These results indicated that, in Williams 82, GmphyA and GmphyB act as functional photoreceptors and nuclear importers, whereas in the *Gmlin1-1* mutant, the nuclear transport of GmphyA and GmphyB is inhibited. Thus, mutations in GmHY2a may prevent the light-inducible photoconversion of Pr to the physiologically active Pfr form, leading to changes in phytochrome signalling transduction in the nucleus.

### The *Gmlin1* mutant exhibited constitutive SARs, causing internode elongation

**Fig. 6.**
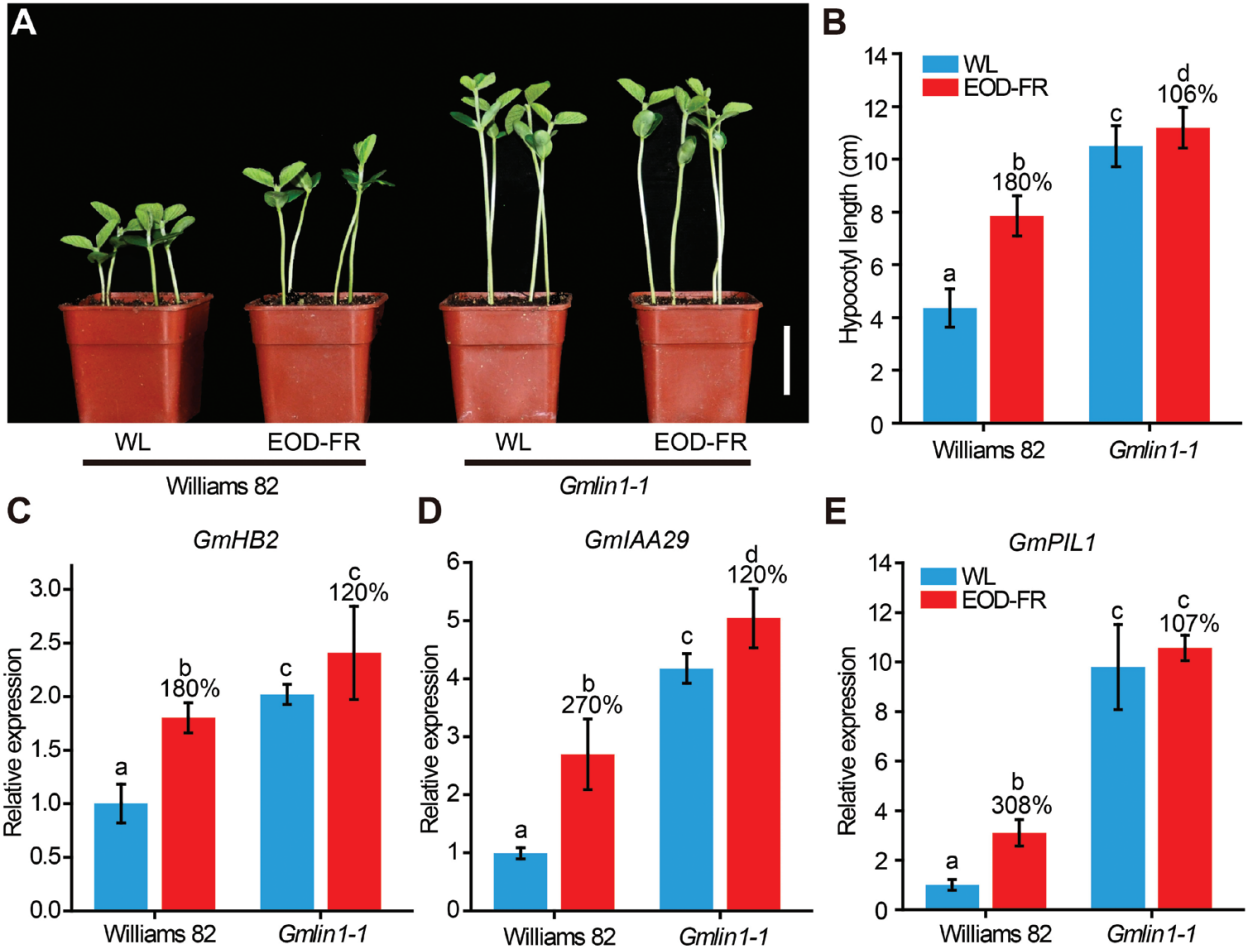
Effects of EOD-FR treatment on Williams 82 and *Gmlin1-1* seedlings. (A) Phenotypes of Williams 82 and *Gmlin1-1* under normal white light (WL, 100 μmol m^−2^ s^−1^) or subjected to EOD-FR treatment (exposure to 14 μmol m^−2^ s^−1^ FR light for 30 min at the end of each light period). Scale bar=5 cm. (B) Hypocotyl lengths. All values are given as means ±SD (n=20). The percentage on the columns represents the hypocotyl length relative to the WL. (C, D, E) RT-qPCR quantification of the relative expression levels of soybean genes associated with the shade response in the seedling hypocotyl. (C) *GmHB2.* (D) *GmIAA29.* (E) *GmPIL1*. All data shown are means ±SD of three biological replicates. Different lowercase letters indicate statistically significant differences (*P*<0.05, Student’s *t*-test).

We next investigated the response of the *Gmlin1-1* mutant to EOD-FR treatment, which mimics shade conditions (i.e. reduced R:FR ratios) and causes similar changes in plant phenotype (Smith and Whitelam, 1997; Takemura *et al.*, 2015). Williams 82 seedlings reacted strongly to simulated shade conditions (EOD-FR), developing significantly elongated hypocotyls (Fig. 6A, B). In contrast, the response of the *Gmlin1-1* seedlings to EOD-FR was greatly reduced, and the difference in hypocotyl length between the seedlings grown under WL and under EOD-FR was slight (Fig. 6A, B). RT-qPCR analysis showed that the differences in the expression levels of three soybean genes (*GmHB2*, *GmIAA29*, and *GmPIL1*), which are homologues of known shade-induced markers (Yang and Li, 2017), were more marked between WL and EOD-FR in Williams 82 than in *Gmlin1-1* (Fig. 6C-E). Under normal WL, the expression levels of three marker genes were significantly increased in the *Gmlin1-1* mutant when compared to Williams 82 (Fig. 6C-E). These results suggested that *Gmlin1* mutants might express a constitutive shade avoidance response, even under normal growth conditions. Moreover, they were less sensitive to shade.

Key phytohormones reported to be involved in the plant response to shading based on an increase in hypocotyl and internode length are gibberellin (GA) and auxin [indole-3-acetic acid (IAA)] (Yang and Li, 2017; Liu *et al.*, 2021). RT-qPCR was performed to measure the relative expression of known IAA-related genes, including *GmIAA9* and *GmSAUR* (*Small Auxin Up RNA*), as well as GA-related genes, including *GmGA20ox* (*GA-20 oxidase*) and *GmGA3ox* (*GA-3 oxidase*), in Williams 82 and the *Gmlin1-1* mutant. Compared with Williams 82, these genes were significantly up-regulated in the *Gmlin1-1* mutant, especially *GmSAUR* and *GmGA3ox* (Supplementary Fig. S6). These results suggested that mutation in GmHY2a might affect the levels of GA and IAA, resulting in promoting internode elongation of the *Gmlin1* mutant.

### Photoperiod sensitivity was reduced and flowering time was accelerated in the *Gmlin1* mutant

**Fig. 7.**
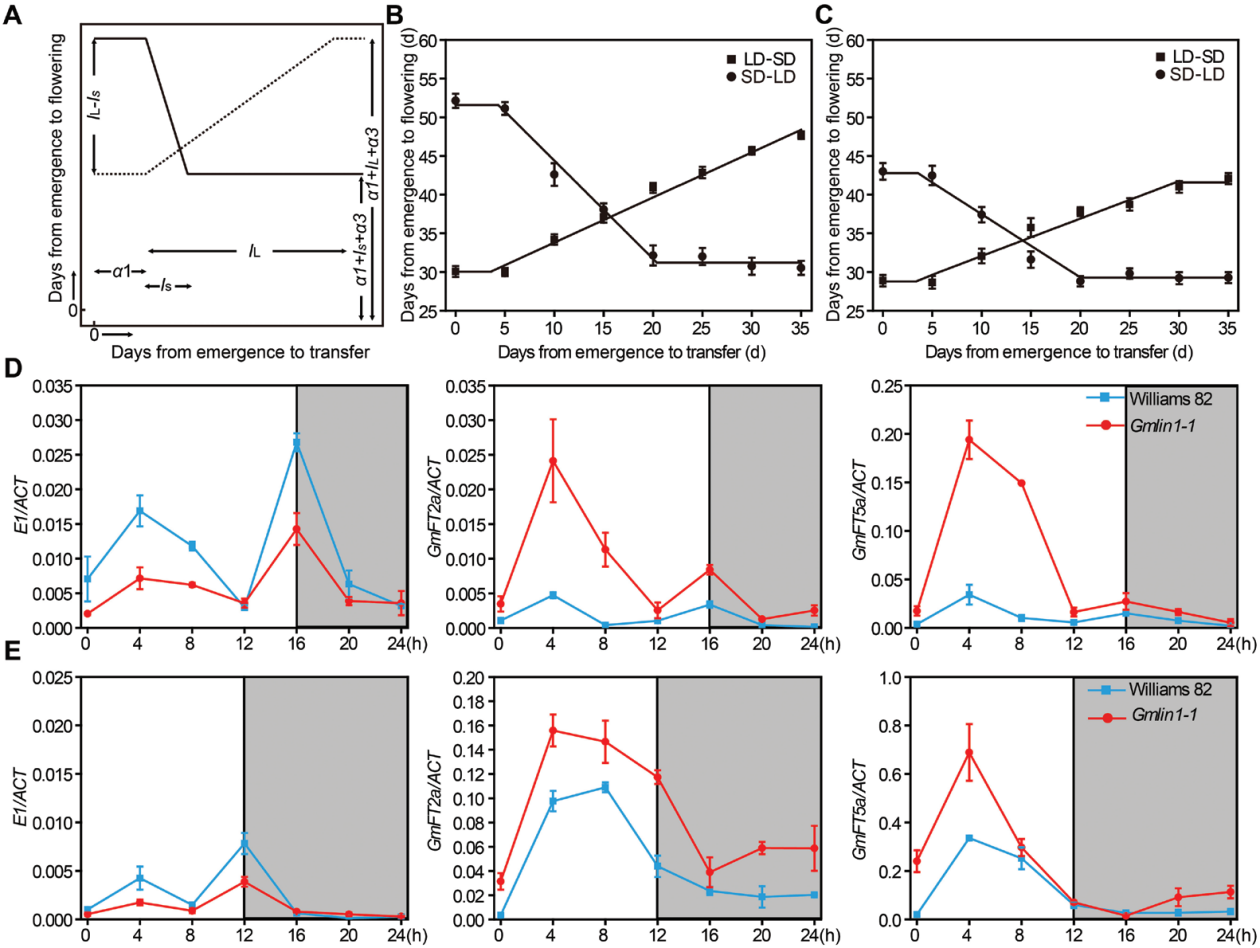
Photoperiodic response of Williams 82 and *Gmlin1-1*. (A) Schematic representation of flowering responses of plants transferred from LD conditions (16 h light/ 8 h dark) to SD conditions (12 h light/ 12 h dark) or vice versa at various times after emergence. Solid lines represent transfers from SD to LD; dashed lines represent transfers from LD to SD. (B) Flowering response of Williams 82. (C) Flowering response of *Gmlin1-1*. All data are means ±SD (n=15). (D, E) Diurnal expression levels of *E1*, *GmFT2a*, *GmFT5a* in Williams 82 and *Gmlin1-1* seedlings grown under (D) LD conditions and (E) SD conditions. Light periods are unshaded; dark periods are shaded. All data are means ±SD of three biological replicates.

To analyse the photoperiod sensitivity of the *Gmlin1-1* mutant, we performed a photoperiod transfer experiment following the model of Ellis *et al.* (1992). Development before floral initiation in soybean consists of three phases: the pre-inductive phase (*a1*), the inductive (photoperiod-sensitive) phase under LD or SD conditions (*I*_L_/ *I*_S_), and the post-inductive phase (*a3*) (Fig. 7A) (Ellis *et al.*, 1992). Segmented linear regression analysis of the flowering times of Williams 82 and *Gmlin1-1* revealed that the duration of the photoperiod-sensitive phase under SD conditions (*I*_S_) was 16 DAE and 15 DAE, respectively, whereas the duration of the photoperiod-sensitive phase under LD conditions (*I*_L_) was 37 DAE and 29 DAE, respectively (Fig. 7B, C). The slope coefficient of the regression line, which reflects photoperiod sensitivity, was lower for the *Gmlin1-1* mutant (*I*_*S*_ slope, -0.89; *I*_*L*_ slope, 0.48) when compared to Williams 82 (*I*_*S*_ slope, -1.27; *I*_*L*_ slope, 0.58), suggesting that the *Gmlin1-1* mutant was less sensitive to photoperiod than Williams 82 (Fig. 7B, C; Supplementary Table S3).

We then measured the expression levels of key genes in the soybean photoperiodic pathway under LD and SD conditions. *E1*, which is the central gene in the soybean photoperiodic pathway (Xia *et al.*, 2012), exhibited a bimodal expression pattern and was down-regulated in *Gmlin1-1* compared to Williams 82 under both LD and SD conditions (Fig. 7D, E). In Williams 82, the transcriptional levels of *GmFT2a* and *GmFT5a*, two flowering inducers in soybean (Kong *et al.*, 2010, 2014), peaked 4 h after dawn and at dusk under LD conditions (Fig. 7D), whereas under SD conditions, these genes were up-regulated during the day and down-regulated at night (Fig. 7E). In general, both genes were up-regulated in Williams 82 under SD conditions compared to LD conditions (Fig. 7D, E). Although the expression patterns of *GmFT2a* and *GmFT5a* in the *Gmlin1-1* mutant were similar to those in Williams 82, both genes were strongly up-regulated in *Gmlin1-1* compared to Williams 82 irrespective of day length (Fig. 7D, E). *GmHY2a* expression was seen throughout the day and night, with a slight increase at 4 h after dawn, then decreased gradually during daytime and increased gradually at night under both SD and LD conditions in Williams 82. The expression levels in the *Gmlin1-1* mutant were slightly lower, but have similar patterns of expression to that of Williams 82 (Supplementary Fig. S7). These results suggest that *GmHY2a* might affect the soybean photoperiodic pathway, and that the downregulation of *E1* in the *Gmlin1* mutant may lead to the upregulation of *GmFT2a* and *GmFT5a* and the consequent acceleration of flowering.

### No significant effect on yield between Williams 82 and the *Gmlin1* mutant

**Fig. 8.**
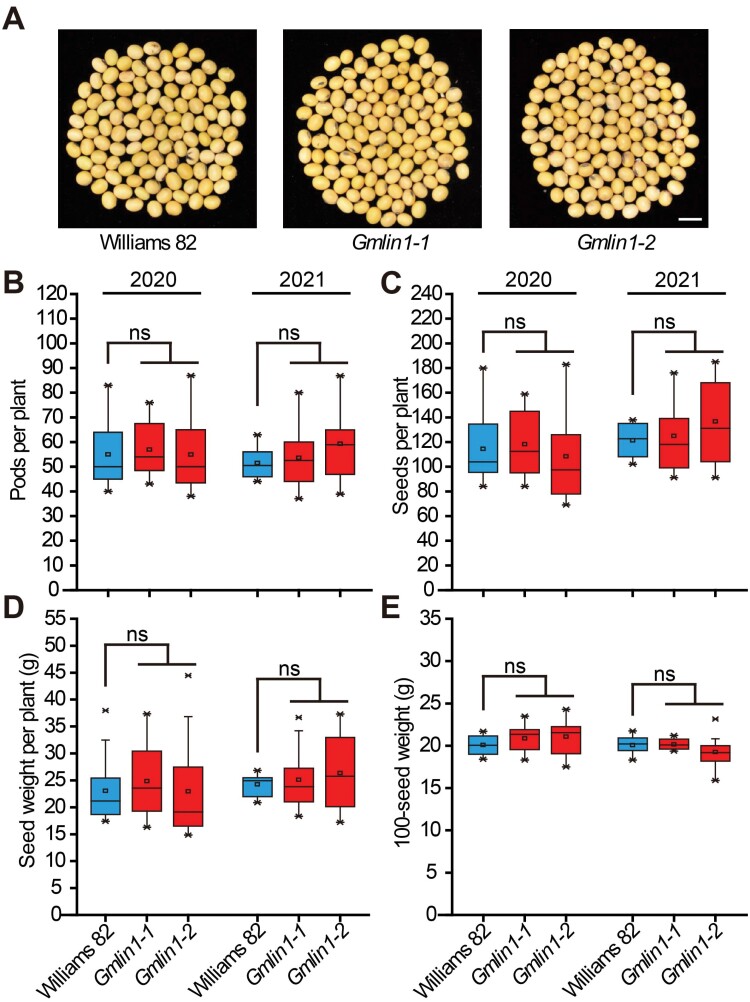
Yield indices of Williams 82 and the *Gmlin1* mutants. (A) Seed phenotypes of Williams 82, *Gmlin1-1*, and *Gmlin1-2*. Scale bar=1 cm. (B-E) Comparison of (B) pods per plant, (C) seeds per plant, (D) seed weight per plant, and (E) 100-seed weight among Williams 82, *Gmlin1-1*, and *Gmlin1-2* grown under natural conditions in the field at Changchun (May to October in 2020 and 2021). All data are means ±SD (at least 10 plants). ns, no significant difference.

Next, we tested whether the *Gmlin1* mutation affected soybean yield. At maturity, seeds of the *Gmlin1-1* and *Gmlin1-2* mutants were of the same size as those of Williams 82 (Fig. 8A). We measured several yield-related parameters viz., pods per plant, seeds per plant, seed weight per plant, and 100-seed weight for Williams 82 and the two *Gmlin1* mutants under normal condition in the field for 2 years, and we found no significant differences in yield-related parameters between Williams 82 and the *Gmlin1* mutants (Fig. 8B-E). These observations indicated that the *Gmlin1* mutation had limited influence on yield at higher latitudes.

## Discussion

### Mutation in soybean GmHY2a dysregulated the phytochrome response to light

Phytochromes are important photoreceptors that respond to ambient light to regulate plant growth and development (Franklin and Quail, 2010). In this study, we found that the long internodes and early flowering of the mutant line *Gmlin1* were associated with the mutation of a single recessive gene, *GmHY2a*, which encodes the PΦB synthase known to be a key enzyme of phytochrome chromophore biosynthesis (Kohchi *et al.*, 2001). Mutations that disrupt PΦB synthase function have been characterized in many species. The first identified and located mutant was Arabidopsis *hy2*, which expresses a long-hypocotyl phenotype and was shown to lack light-reversible phytochrome (Koornneef *et al.*, 1980; Kohchi *et al.*, 2001). Subsequently, *hy2* mutants have been identified in many common crops, including tomato (*aurea*; Muramoto *et al.*, 2005), maize (*elm1*; Sawers *et al.*, 2002, 2004), rice (*se13*; Saito *et al.*, 2011; Yoshitake *et al.*, 2015), and cucumber (*elh1*; Hu *et al.*, 2021). These mutants, which are defined as phytochrome chromophore-deficient mutants, have similar phenotypes that are consistent with severe reductions in functional phytochromes. Similar to these previously reported species, the soybean *Gmlin1* mutant responded weakly to both R and FR light and expressed a photomorphogenesis defective phenotype (Fig. 4). This indicated that, across multiple plant species, GmHY2a is functionally conserved and that HY2 proteins play a role in phytochrome biosynthesis. In the rice mutant *se5*, which harbours a mutation in a heme oxygenase important for phytochrome chromophore synthesis, PHYA and PHYB were mostly found in the cytoplasm under light conditions, suggesting that phytochromes could not be effectively imported into the nucleus, where most signalling functions occur (Zheng *et al.*, 2019). Similarly, phytochromes were not effectively imported into the nucleus in the *Gmlin1* mutant exposed to light (Fig. 5). Our results indicated that the entire phytochrome system was inactivated and that the mutation in GmHY2 influenced the conversion of Pr to Pfr, leading to the accumulation of the Pr form in the cytoplasm.

### The *Gmlin1* mutant exhibited constitutive SARs, and this mutation might influence the auxin and gibberellin pathways to promote internode elongation

Previous studies have shown that shade conditions or treatment with low R:FR light leads to the conversion of phytochromes from Pfr to Pr and induces many auxin-responsive genes to increase stem elongation (Müller-Moulé *et al.*, 2016). It has been shown that active phyB is translocated into the nucleus, where it acts to repress SAR by inhibiting the activities of a group of positive regulators of SAR, including PIF3, PIF4, PIF5, and PIF7 (Lorrain *et al.*, 2008). The PIFs fine-tune the SAR by directly targeting core marker genes, including *ATHB2* (*HOMEOBOX2*) and *PIL1* (*PIF-LIKE 1*) (Kunihiro *et al.*, 2011). *ATHB2* is a member of the homeodomain leucine zipper (HDZip) family of transcription factors (Chen *et al.*, 2014; Zhang *et al.*, 2019). In Arabidopsis, *ATHB2* transcript abundance increases rapidly following EOD-FR treatment, leading to the expression of a series of SAR phenotypes (Schena *et al.*, 1993); the basic helix-loop-helix (bHLH) transcription factor *PIL1* is also rapidly up-regulated in Arabidopsis under low R:FR conditions (Hornitschek *et al.*, 2009). *PIL1* is also reported to induce the endogenous auxin biosynthesis pathway in plants exhibiting SAS via auxin signalling components, such as AUX/IAAs (Procko *et al.*, 2016), and auxin has been implicated in hypocotyl and stem elongation in plants (Liscum *et al.*, 2002). Consistent with these previous studies, *GmHB2*, *GmIAA29*, and *GmPIL1* were significantly up-regulated in Williams 82 following EOD-FR treatment (Fig. 6C-E). Under normal WL, all three marker genes were up-regulated in the *Gmlin1* mutant compared to Williams 82 and thus may play an important role in the elongation of the internode and hypocotyl in the *Gmlin1* mutant. Up-regulation of the auxin-responsive genes *GmIAA29*, *GmIAA9*, and *GmSAUR* (*Small Auxin Up RNA*) in the *Gmlin1* mutant suggested that one mechanism underlying the expression of the long internode phenotype in the mutant may be the promotion of auxin synthesis (Fig. 6D; Supplementary Fig. S6A, B). In addition, gibberellin also plays a major role in shade-induced elongation of the hypocotyl and stem. It has been reported that PIFs are released from repression by Pr-form phytochromes, increasing GA levels (Liu *et al.*, 2021). Low R/FR conditions up-regulate the GA synthesis genes *GA20ox1* and *GA20ox2* (Hisamatsu *et al.*, 2005). In a recent report, soybean *GmGA3ox* was found to be functionally conserved in GA biosynthesis regulating plant height and yield (Hu *et al.*, 2022). Here, the *GmGA20ox* and *GmGA3ox* genes were significantly up-regulated in the *Gmlin1* mutant, suggesting that GA was also involved in hypocotyl and internode elongation of the *Gmlin1* mutant (Supplementary Fig. S6C, D). Together, our results showed that mutations in GmHY2a prevent the phytochrome-mediated regulation of downstream genes in the nucleus and increase the expression of auxin- and gibberellin-related genes to promote internode elongation.

### The early flowering and reduced photoperiod sensitivity of *Gmlin1* indicate that this mutant might successfully tolerate LD conditions at higher latitudes

In soybean, flowering time is an important agronomic trait that determines regional adaptation and yield. The widespread latitudinal expansion of soybean depends on reducing photoperiod sensitivity in order to adapt to the LD conditions at higher latitudes (Lin *et al.*, 2021). The GmHY2 mutation affected all soybean holo-phytochromes, and the *Gmlin1* mutant flowered earlier than Williams 82 under both LD and SD conditions; flowering was particularly early under LD conditions (Fig. 1C). To compare the photoperiod sensitivity of Williams 82 and the *Gmlin1* mutant, we performed reciprocal transfer experiments between the LD and SD photoperiods. In chickpeas, similar photoconversion experiments demonstrated that time to flowering is positively correlated with the duration of photoperiod sensitivity: plants with later flowering times have longer periods of photosensitivity, and plants with earlier flowering times have shorter periods of photosensitivity (Daba *et al.*, 2016). Consistent with this, the photoperiod-sensitive phase of the *Gmlin1* mutant was shorter than that of Williams 82 under both LD and SD conditions (Fig. 7B, C; Supplementary Table S3). In addition, based on the slope coefficients of the flowering responses, which can be used to estimate photoperiod sensitivity, the *Gmlin1* mutant was less sensitive to photoperiod than Williams 82 (Fig. 7B, C; Supplementary Table S3).

Phytochromes are photoreceptors that mediate light signals to regulate the photoperiodic response and control flowering time (Franklin and Quail, 2010). The soybean *E3* and *E4* genes, which encode homologues of phytochrome A (GmphyA3 and GmphyA2, respectively), are known photoreceptors in the soybean photoperiodic response (Liu *et al.*, 2008; Watanabe *et al.*, 2009). In the double recessive and non-functional *e3/e4* genotype soybean cultivar, early flowering was observed under both natural day length and artificial LD conditions (Xu *et al.*, 2013; Lin *et al.*, 2021). Similarly, we demonstrated that *Gmlin1*, which lacks all phytochromes, also exhibited early flowering under LD and SD conditions. The photoperiod insensitivity and early flowering of the *Gmlin1* mutants were reflected in the expression patterns of genes involved in the photoperiod regulatory pathway. The expression of *E1* was also suppressed in the *Gmlin1* mutant, as in the soybean *e3*/*e4* genotype (Xu *et al.*, 2015). Early flowering is often associated with a short vegetative stage, leading to reduced soybean yield. Our results indicated that the GmHY2a mutation does not reduce weight per plant or decrease grain number per plant (Fig. 8), with *Gmlin1* exhibiting a WT-like yield per plant. We identified two possible explanations for this result: first, the constitutive shade avoidance phenotype of the *Gmlin1* mutant may have led to the growth of petioles or branches at more acute angles increasing sunlight capture and compensating for decreases in photosynthetic efficiency. Alternatively, the North American cultivar Williams 82, which is suitable for cultivation in the Huanghuai region of China, may not have been the most suitable choice for cultivation at higher latitudes, resulting in misleadingly low yields. A premise of soybean adaptation to long-day conditions at higher latitudes is that these adaptations should not affect normal growth. To sum up, the photoperiod-insensitive mutant *Gmlin1* may represent a useful reference point for soybean cultivation under LD conditions at higher latitudes.

## Supplementary data

The following supplementary data are available at *JXB* online.

Fig. S1. Sequence analysis of the mutation site in *GmHY2a* in *Gmlin1-1*, *Gmlin1-2*, and F_1_ (*Gmlin1-1*×*Gmlin1-2*) plant.

Fig. S2. Bulked segregant analysis (BSA) mapping of *Gmlin1-1*.

Fig. S3. The transcription sequence of *GmHY2a* in Williams 82 and *Gmlin1-1*.

Fig. S4. Alignment of GmHY2 and known homologous proteins from *Lycopersicon esculentum*, *Glycine max*, *Arabidopsis thaliana*, *Zea mays*, *Oryza sativa*, and *Cucumis sativus*.

Fig. S5. Comparison of structure and function between GmHY2a and GmHY2b.

Fig. S6. Relative expression levels of plant height-related genes in Williams 82 and *Gmlin1-1*.

Fig. S7. Diurnal expression levels of *GmHY2a* in Williams 82 and *Gmlin1-1*.

Table S1. The primers used in this study.

Table S2. Ka/Ks values and estimation of the absolute dates of *GmHY2* gene duplications.

Table S3. Sub-phase length in plants transferred from SD to LD or LD to SD conditions.

erac318_suppl_Supplementary_Material

## Data Availability

The sequencing datasets generated during and/or analysed during the current study are available in the Genome Sequence Archive (GSA) database in the BIG Data Center (https://bigd.big.ac.cn/gsa/index.jsp) under accession number CRA005967.

## Abbreviations

BSA: bulked segregant analysis
BV: biliverdin
DAE: days after emergence
EMS: ethyl methyl sulfonate
EOD-FR: end-of-day far-red
FDBR: ferredoxin-dependent biliverdin reductase
FR: far-red light
*Gmlin1*: *Glycine max long internode 1*
Ka: non-synonymous substitutions per non-synonymous site
Ks: synonymous substitutions per synonymous site
LD: long-day
PΦB synthase: phytochromobilin synthase
R: red light
RT-qPCR: real time quantitative PCR
SAS: shade avoidance syndrome
SAR: shade avoidance response
SD: short-day
SNPs: single-nucleotide polymorphisms
YFP: yellow fluorescent protein
