# Supplementary material for: Soybean GmHY2a encodes a phytochromobilin synthase that regulates internode length and flowering time
Source: J Exp Bot. 2022 Aug 10;73(19):6646–62. doi: 10.1093/jxb/erac318 (PMC9629791; doi:10.1093/jxb/erac318)
Supplement: erac318_suppl_Supplementary_Material [file erac318_suppl_supplementary_material.pdf]

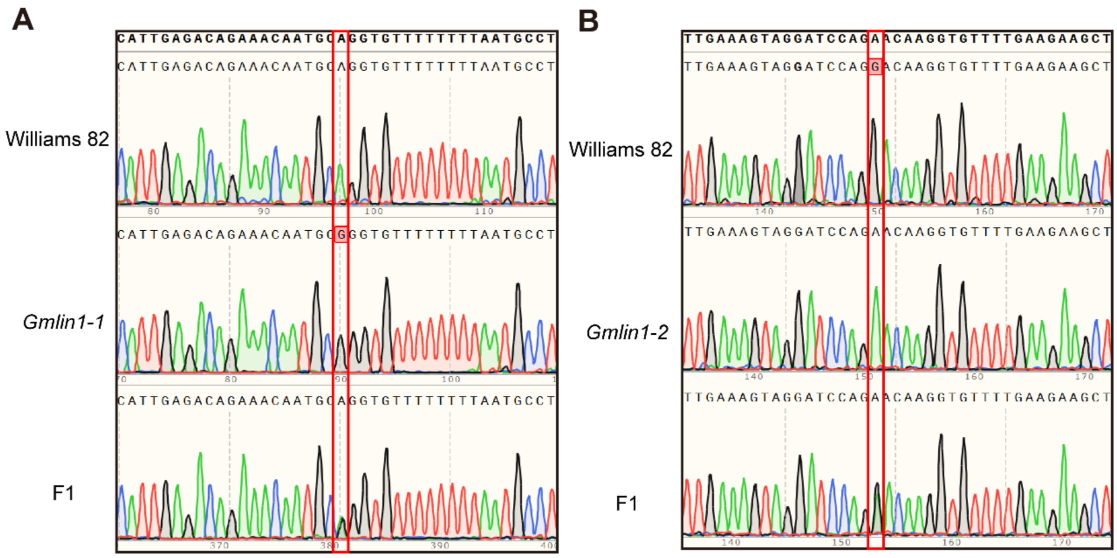

Fig. S1 Sequence analysis of the mutation site in GmHY2a in *Gmlin1-1*, *Gmlin1-2*, and the F_1 (*Gmlin1-1*×*Gmlin1-2*) plant. **(A)** The mutation site of the *Gmlin1-1* mutant. **(B)** The mutation site of the *Gmlin1-2* mutant.

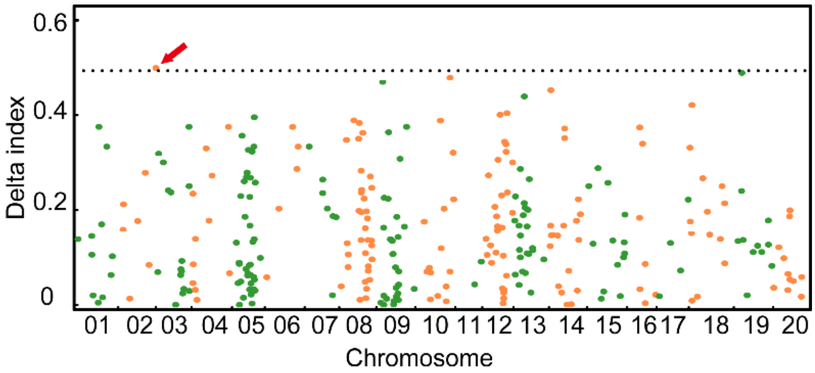

Fig. S2 Bulked segregant analysis (BSA) mapping of *Gmlin1-1*. The delta SNP index is plotted over all chromosomes of the M_2 population of the *Gmlin1-1* mutant.

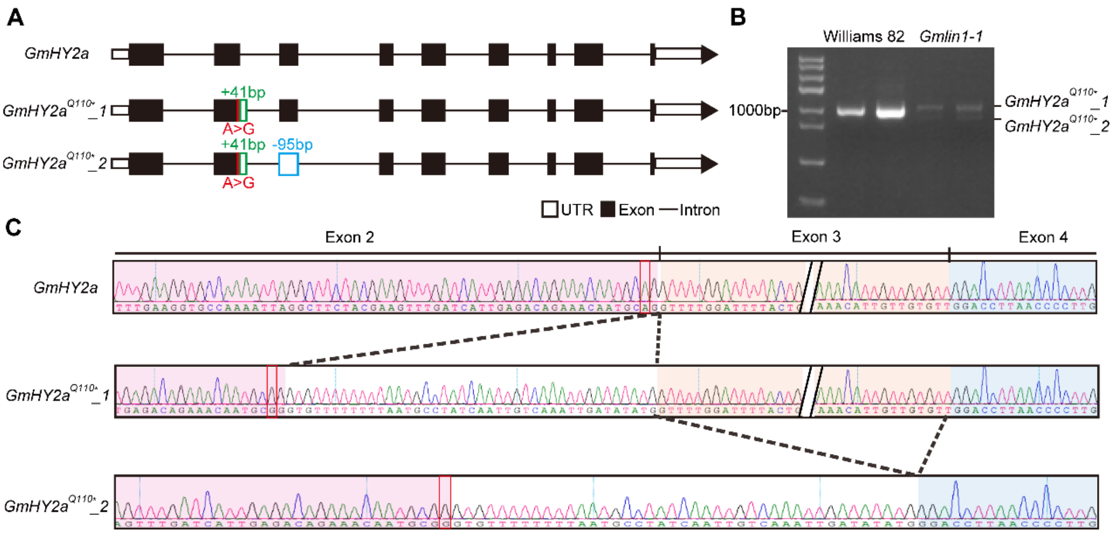

Fig. S3 The transcription sequence of *GmHY2a* in Williams 82 and *Gmlin1-1*. **(A)** Genomic structures of the wild-type *GmHY2a* and the mutants *GmHY2a*^{Q110*}*_1* and *GmHY2a*^{Q110*}*_2*. The red vertical lines represent mutated sites. The green squares represent insertions, while the blue square represents exon loss. **(B)** PCR amplification of *Glyma.02G304700* from Williams 82 and *Gmlin1-1*. **(C)** Chromatograms of the coding sequences of Williams 82 and the mutants *GmHY2a*^{Q110*}*_1* and *GmHY2a*^{Q110*}*_2*. Exon 2, exon 3, and exon 4 are shaded light purple, orange, and blue, respectively. Red boxes indicate the location of the mutations in *Gmlin1-1*. The trace of exon 3 is fractured. Lost and inserted sequences are identified using dashed lines.

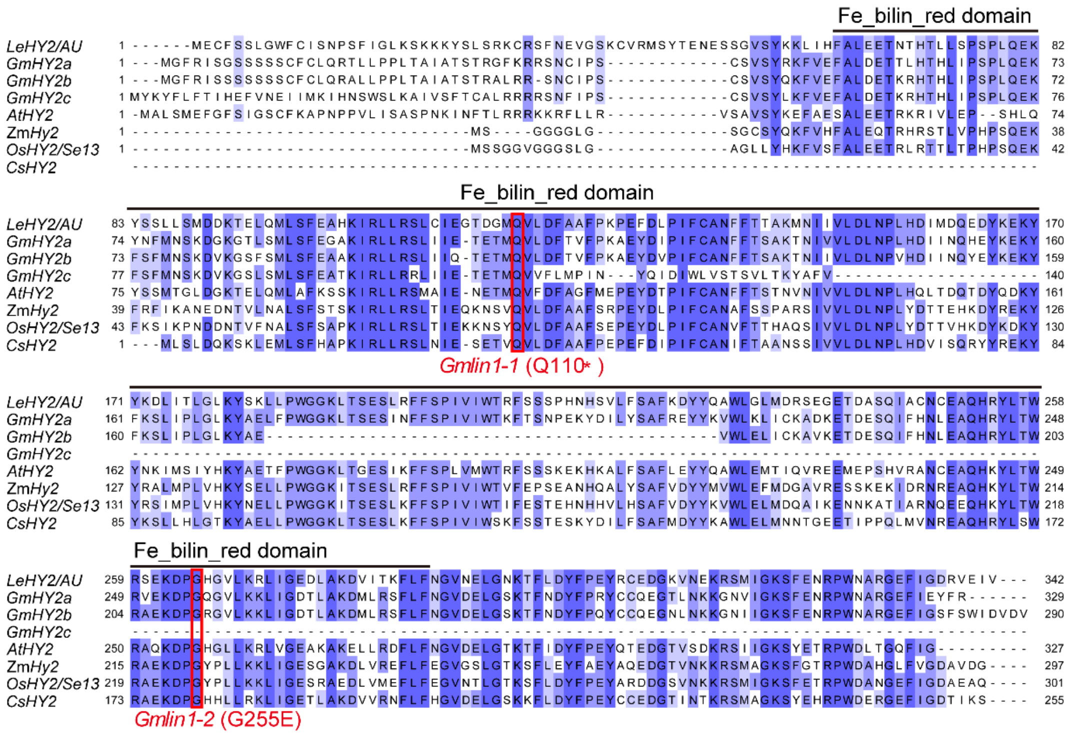

Fig. S4 Alignment of GmHY2 and known homologous proteins from *Lycopersicon esculentum*, *Glycine max*, *Arabidopsis thaliana*, *Zea mays*, *Oryza sativa*, and *Cucumis sativus*. Protein sequences were aligned using Clustal W and generated using Jalview 2.10.3. Amino acid residues were color-coded based on the BLOSUM62 score. Red boxes indicate the mutation locations in the two allelic mutants (*Gmlin1-1* and *Gmlin1-2*).

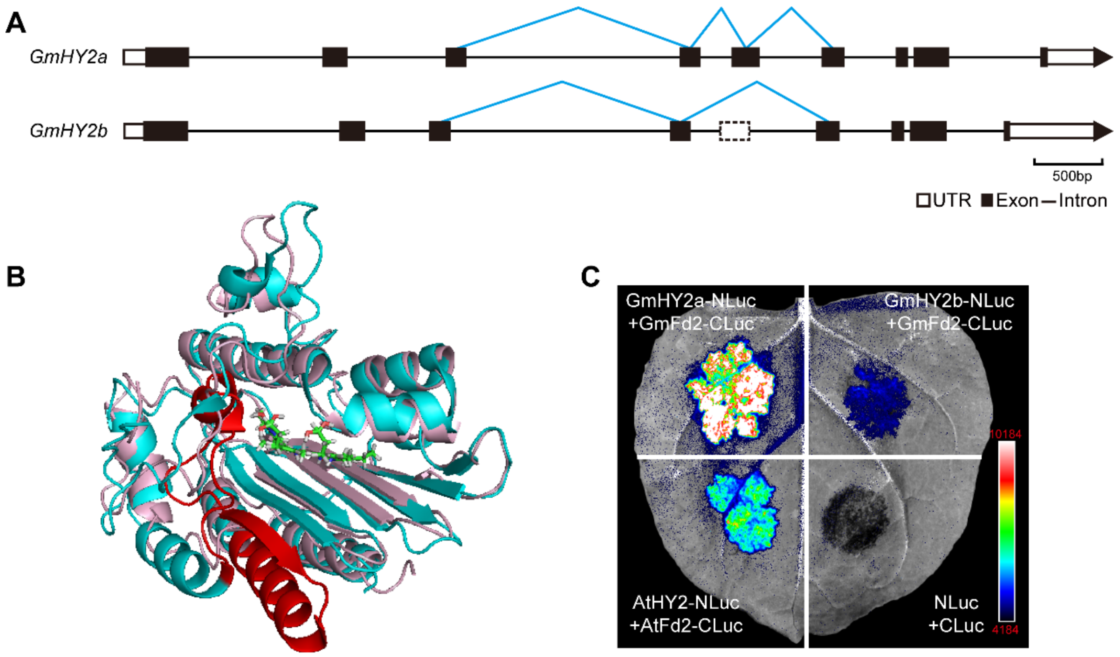

Fig. S5 Comparison of structure and function between GmHY2a and GmHY2b. **(A)** Genomic structures of *GmHY2a* and *GmHY2b*. The dotted boxes represent the missing exon of *GmHY2b*. The blue lines represent alternative splicing. **(B)** GmHY2b protein 3D structure (pink) and merged with the known LeHY2 of *Lycopersicon esculentum* (cyan). The red areas represent GmHY2b missing areas. Stick represents substrate BV IXα **(C)** Interactions between GmHY2 and GmFd2 in luciferase complementation assays (LCIs) performed in *Nicotiana benthamiana* leaves. The interaction between AtHY2 and AtFd2 (the Arabidopsis homologous of GmHY2 and GmFd2, respectively) was used as the positive control. NLUC and CLUC correspond to the luciferin N- and C-termini, respectively.

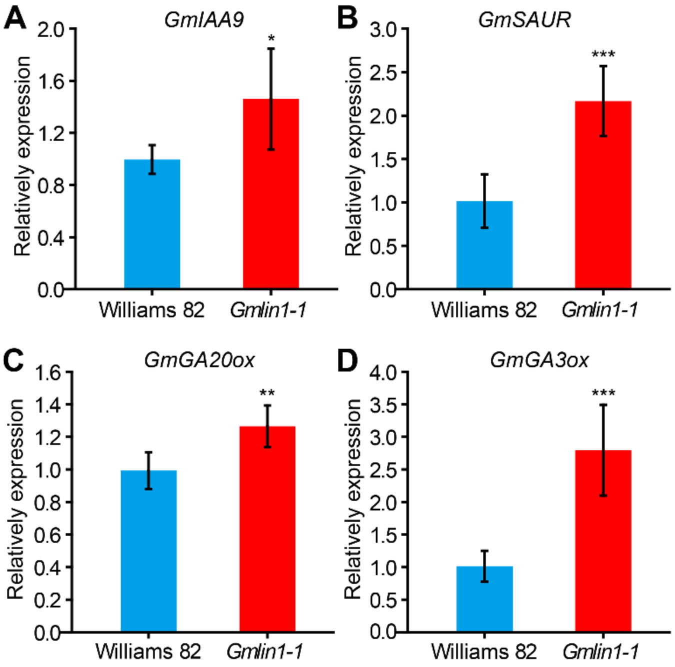

Fig. S6 Relative expression levels of plant height-related genes in Williams 82 and *Gmlin1-1*. RT-qPCR quantification of the relative expression levels of the IAA-signaling genes **(A)** *GmIAA9* and **(B)** *GmSAUR*, as well as the GA-biosynthesis genes **(C)** *GmGA20ox* and **(D)** *GmGA3ox*. Expression levels are shown as means ± SD. Asterisk indicate statistically significant differences (***P < 0.001, **P < 0.01, *P < 0.05, Student's *t* test).

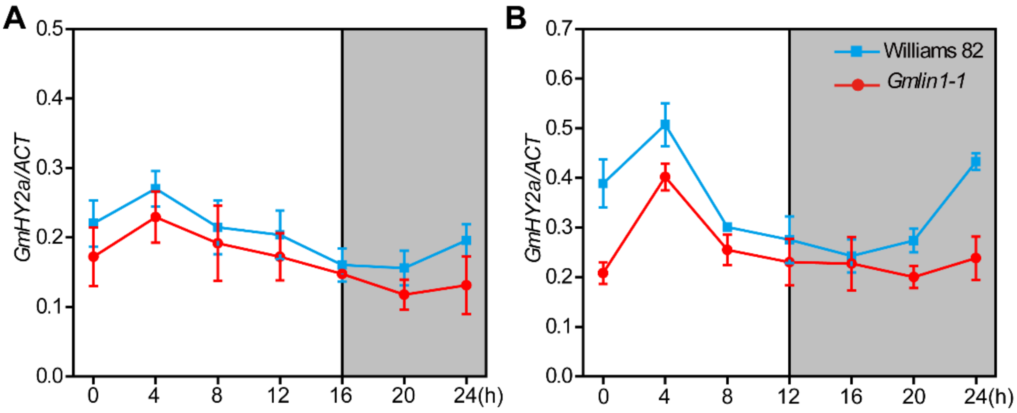

Fig. S7 Diurnal expression levels of *GmHY2a* in Williams 82 and *Gmlin1-1* seedlings grown under **(A)** LD conditions and **(B)** SD conditions. Fully expanded trifoliate leaves were sampled every 4 h starting at dawn at 15 DAE. All data are means ± SD. Light periods are unshaded; dark periods are shaded gray.

Table S1. The primers used in this study

Primer name	Primer sequence (5'- 3')	Annotation	gene locus
OL9037 F	ATGGGTTTTAGAATTAGCGGT	CDS *GmHY2a*	*Glyma.02G304700*
OL9038 R	CCTAAAATATTCAATAAATTCTCCTC		
OL11272 F	CAATCACCAAGTCTTGGCTTCTC	*GmHY2a* mutation in *Gmlin1-1*	*Glyma.02G304700*
OL11273 R	TTCCAAATTGACTGATGTTCTCA		
OL8829 F	GCCAATCCTATCAGGACTTT	*GmHY2a* mutation in *Gmlin1-2*	*Glyma.02G304700*
OL8830 R	CCCTCTTGACAGCAGTAGCG		
OL9310 F	CTCAGAATTCGAGCTCGTATTTTGCATACGACGTTTAAG	*pGmHY2a:GmHY2a*	*Glyma.02G304700*
OL9315 R	GTCTAGAGGATCCCGGGACAAAGAGACCCAATTTAGCC		
OL9801 F	CAAAAAAGCAGGCTTCATGGGTTTTAGAATTAGCGGTTCCT	YFP-GmHY2a	*Glyma.02G304700*
OL9802 R	CAAGAAAGCTGGGTCCCTAAAATATTCAATAAATTCTCCT		
OL10947 F	ACGGGGGACGAGCTCGGTACCATGGCTTCCTCTATGCTCTCTTC	RbcS-mCherry	*AT1G67090*
OL10948 R	GCCCTTGCTCACCATGTCGACACCGGTGAAGCTTGGTGG		
OL12616 F	CGAACGATACTCGAGGTCGACATGTCCTCTTCAAGGCCCAG	GmPHYA-YFP	*Glyma.19G224200*
OL12617 R	CATACTAGTGGATCCCCCGGGAGTGTTGGATTTATGTGCTGCGG		
OL12618 F	CGAACGATACTCGAGGTCGACATGGCTTCAGCAAGCGGAG	GmPHYB-YFP	*Glyma.09G035500*
OL12619 R	CATACTAGTGGATCCCCCGGGACACTTTTTAGAGCTTCTCCGTG		
OL11082 F	ACGGGGGACGAGCTCGGTACCATGGCTTTATCAATGGAGTTTGGG	AtHY2-NLUC	*AT3G09150*
OL11083 R	AACATCGTATGGGTAGTCGACGCCGATAAATTGTCCTGTTAAATCC		
OL11084 F	ACGGGGGACGAGCTCGGTACCATGGCTTCCACTGCTCTCTCAAG	AtFd2-CLUC	*AT1G60950*
OL11085 R	CGCGTACGAGATCTGGTCGACAACAATGTCTTCTTCTTTGTGGGTT		
OL10558 F	ACGGGGGACGAGCTCGGTACCATGGGTTTTAGAATTAGCGGT	GmHY2a-NLUC	*Glyma.02G304700*
OL10559 R	AACATCGTATGGGTAGTCGACCCTAAAATATTCAATAAATTCTCCTC		

OL11086 F	ACGGGGGACGAGCTCGGTACCATGGGATTTAGAATTAGCAGTTCCT	GmHY2b-NLUC	*Glyma.14G009100*
OL11087 R	AACATCGTATGGGTAGTCGACCACATCAACGTCAATCCAGCTG		
OL11090 F	ACGGGGGACGAGCTCGGTACCTTCATCGTGCATTCAATGGCC	GmFd2-CLUC	*Glyma.05G168400*
OL11091 R	CGCGTACGAGATCTGGTCGACTTCGATCTCTCCCTCCTTGTGTGT		
OL9741 F	CCACTCGTGGCTTCAAGAG	*GmHY2a-qPCR*	*Glyma.02G304700*
OL9742 R	CCTGTAAAGGCGAAGGGATC		
OL9907 F	ATCTGACATGGAGAGCTGAAAAG	*GmHY2b-qPCR*	*Glyma.14G009100*
OL9908 R	GCTTCCAAGTTCATCGACTCC		
OL13901 F	CTTGGAACGAGGCTTTCACTTC	*GmHB2-qPCR*	*Glyma.17G144700*
OL13902 R	TTCCACTCACGCTAGAGACGG		
OL13911 F	ACCACACCATGTTATGCTTCCT	*GmIAA29-qPCR*	*Glyma.13G159000*
OL13912 R	TCCTCCAATGGTTTACTGGTG		
OL13915 F	GTGTTGCACGCAGTTTGG	*GmPIL1-qPCR*	*Glyma.10G138800*
OL13916 R	TTGGTGGAATCAGGCATCTT		
OL12902 F	GATAGAGAGACCCTGTGCCT	*GmGA20ox*-qPCR	*Glyma.09G149200*
OL12903 R	TGAGAAGCAGAGCAAAACAGAG		
OL12912F	GCCTCCTCCAAGACATTCAA	*GmGA3ox*-qPCR	*Glyma.15G012100*
OL12913R	AGCCATCAACACCGTCAG		
OL12960 F	CTTGGCCTTCCGGGATCC	*GmIAA9*-qPCR	*Glyma.01G098000*
OL12961 R	GGGAACAGTGGTTTCTCATC		
OL11302F	TTTCCGTTTTCCTGCCATTCG	*GmSAUR*-qPCR	*Glyma.09G220200*
OL11303R	GATATGGGGATCACGAACCTCCTC		
OL10485 F	CACTCAAATTAAGCCCTTTCA	*GmE1-qPCR*	*Glyma.06G207800*
OL10486 R	TTCATCTCCTCTTCATTTTTGTTG		
OL10491 F	ATCCCGATGCACCTAGCCCA	*GmFT2a-qPCR*	*Glyma.16G150700*
OL10492 R	ACACCAAACGATGAATCCCCA		

OL10493 F	TTACAAGCTCCGTTTCTTTGACTG	*GmFT5a-qPCR*	*Glyma.16G044100*
OL10494 R	CCTCAAGACAGGGTTGCTAGG		
OL9129 F	ATCTTGACTGAGCGTGGTTATTCC	*GmACT11-qPCR*	*Glyma.18G290800*
OL9130 R	GCTGGTCCTGGCTGTCTCC		

Table S2. Ka/Ks values and estimation of the absolute dates of *GmHY2* gene duplications

Locus 1	Locus 2	Ka	Ks	Ka/Ks	Date (Mya)	Average Date (Mya)
Glyma.02G304700	*Glyma.14G009100*	0.0461	0.1008	0.4570	8.26	-
Glyma.02G304700	*Glyma.14G136300*	0.3100	0.4437	0.6986	36.37	-
Glyma.14G009100	*Glyma.14G136300*	0.2543	0.3260	0.7800	31.63	-
Glyma.02G304100	*Glyma.14G009700*	0.0164	0.0677	0.2426	5.55	11.15
Glyma.02G304300	*Glyma.14G009500*	0.5444	0.4134	1.3171	33.88	11.15
Glyma.02G304400	*Glyma.14G009400*	0.0954	0.1678	0.5688	13.75	11.15
Glyma.02G304500	*Glyma.14G009300*	0.0094	0.0933	0.1005	7.65	11.15
Glyma.02G304600	*Glyma.14G009200*	0.0351	0.2111	0.1664	17.30	11.15
Glyma.02G304700	*Glyma.14G009100*	0.0461	0.1008	0.4570	8.26	11.15
Glyma.02G304900	*Glyma.14G009000*	0.0235	0.1018	0.2305	8.34	11.15
Glyma.02G305000	*Glyma.14G008900*	0.0161	0.0666	0.2423	5.46	11.15
Glyma.02G305100	*Glyma.14G008300*	0.0034	0.0620	0.0556	5.08	11.15
Glyma.02G305200	*Glyma.14G008200*	0.0220	0.0819	0.2692	6.71	11.15
Glyma.02G305400	*Glyma.14G008000*	0.0050	0.1303	0.0383	10.68	11.15
Glyma.14G134900	*Glyma.17G197800*	0.0343	0.2331	0.1473	19.11	12.40
Glyma.14G135100	*Glyma.17G197600*	0.0091	0.2037	0.0448	16.69	12.40
Glyma.14G135400	*Glyma.17G197500*	0.0249	0.2213	0.1123	18.14	12.40
Glyma.14G135900	*Glyma.17G197200*	0.0375	0.0750	0.4996	6.15	12.40
Glyma.14G136400	*Glyma.17G196900*	0.0204	0.1132	0.1806	9.28	12.40
Glyma.14G136700	*Glyma.17G196800*	0.0025	0.0613	0.0411	5.02	12.40
Glyma.14G136900	*Glyma.17G196700*	0.0239	0.1453	0.1644	11.91	12.40
Glyma.14G137100	*Glyma.17G196400*	0.0192	0.1576	0.1216	12.92	12.40

Ks (non-synonymous nucleotide substitution rate); **Ka** (synonymous nucleotide substitution rate); **MYA** (million years ago)

Table S3. Sub-phase length in plants transferred from SD to LD or LD to SD conditions

**Genotype**	**Transfer**	***a*1 (DAE)**	**I_S/I_L (DAE)**	***a*3 (DAE)**	**I_S/I_L slope**	**R^2**
Williams 82	SD-LD	4.34	16.04	9.64	-1.27	0.99
	LD-SD	3.53	36.96	11.09	0.58	0.99
Gmlin1-1	SD-LD	3.98	15.09	9.70	-0.89	0.97
	LD-SD	3.21	29.64	13.13	0.48	0.95
